# Supplementary figures and images for: A feed-forward pathway drives LRRK2 kinase membrane recruitment and activation
Source: eLife. 2022 Sep 23;11:e79771. doi: 10.7554/eLife.79771 (PMC9576273; doi:10.7554/eLife.79771)

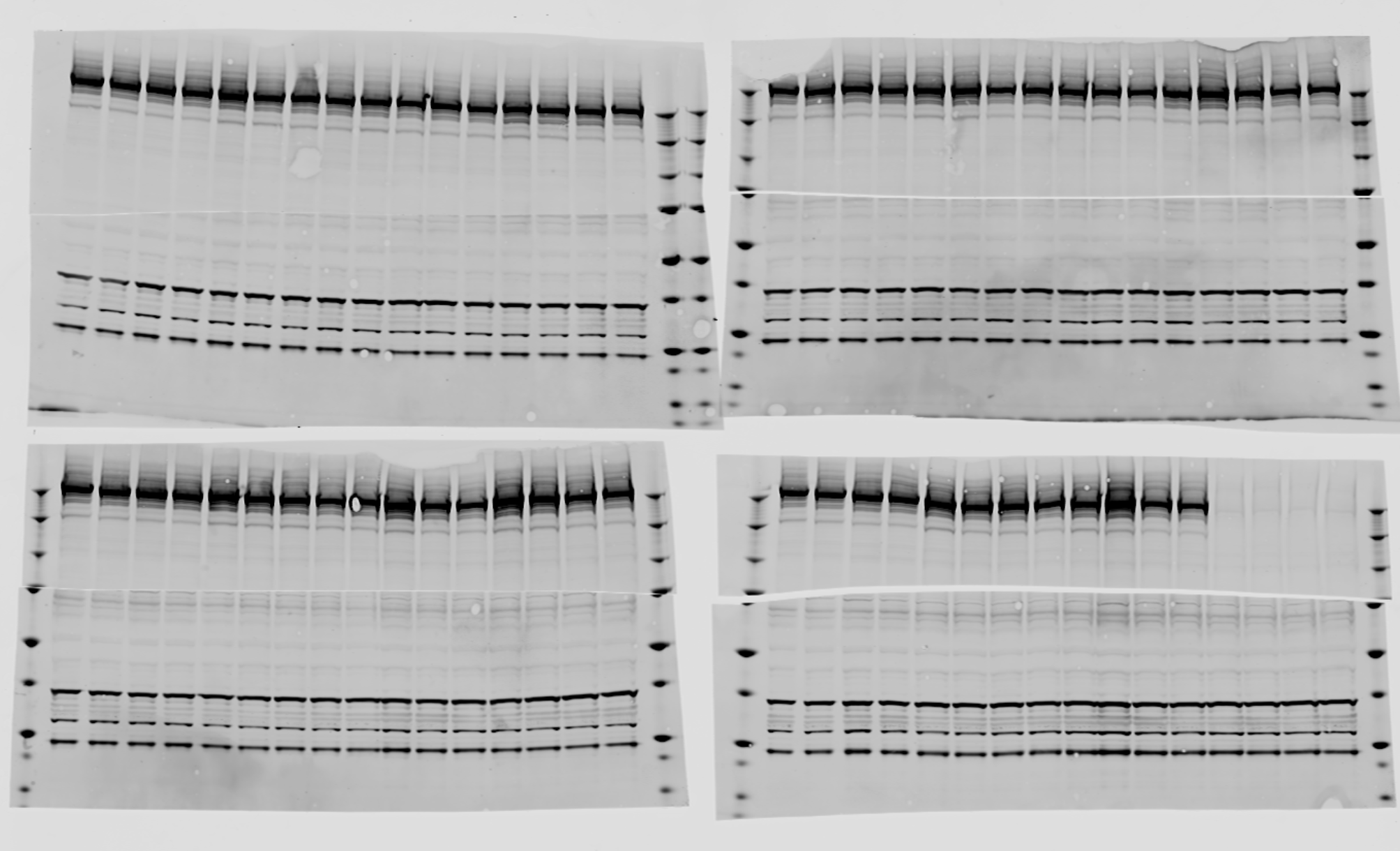

Supplement: Figure 3—figure supplement 4—source data 1. [file elife-79771-fig3-figsupp4-data1.zip › IB raw data/Fig.3.S4A_700(tRab10;tLRRK2)_High.tif]

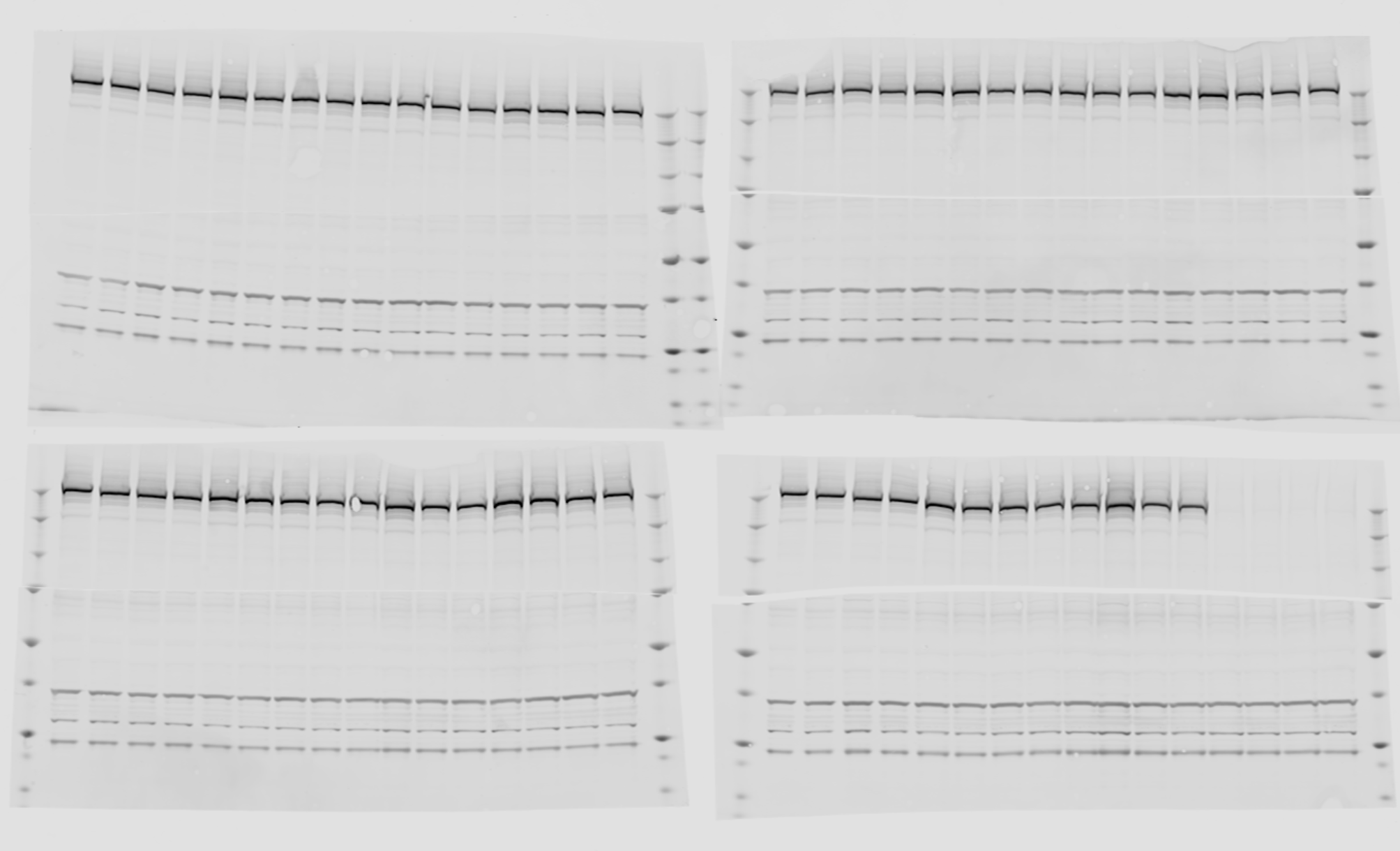

Supplement: Figure 3—figure supplement 4—source data 1. [file elife-79771-fig3-figsupp4-data1.zip › IB raw data/Fig.3.S4A_700(tRab10;tLRRK2)_Low.tif]

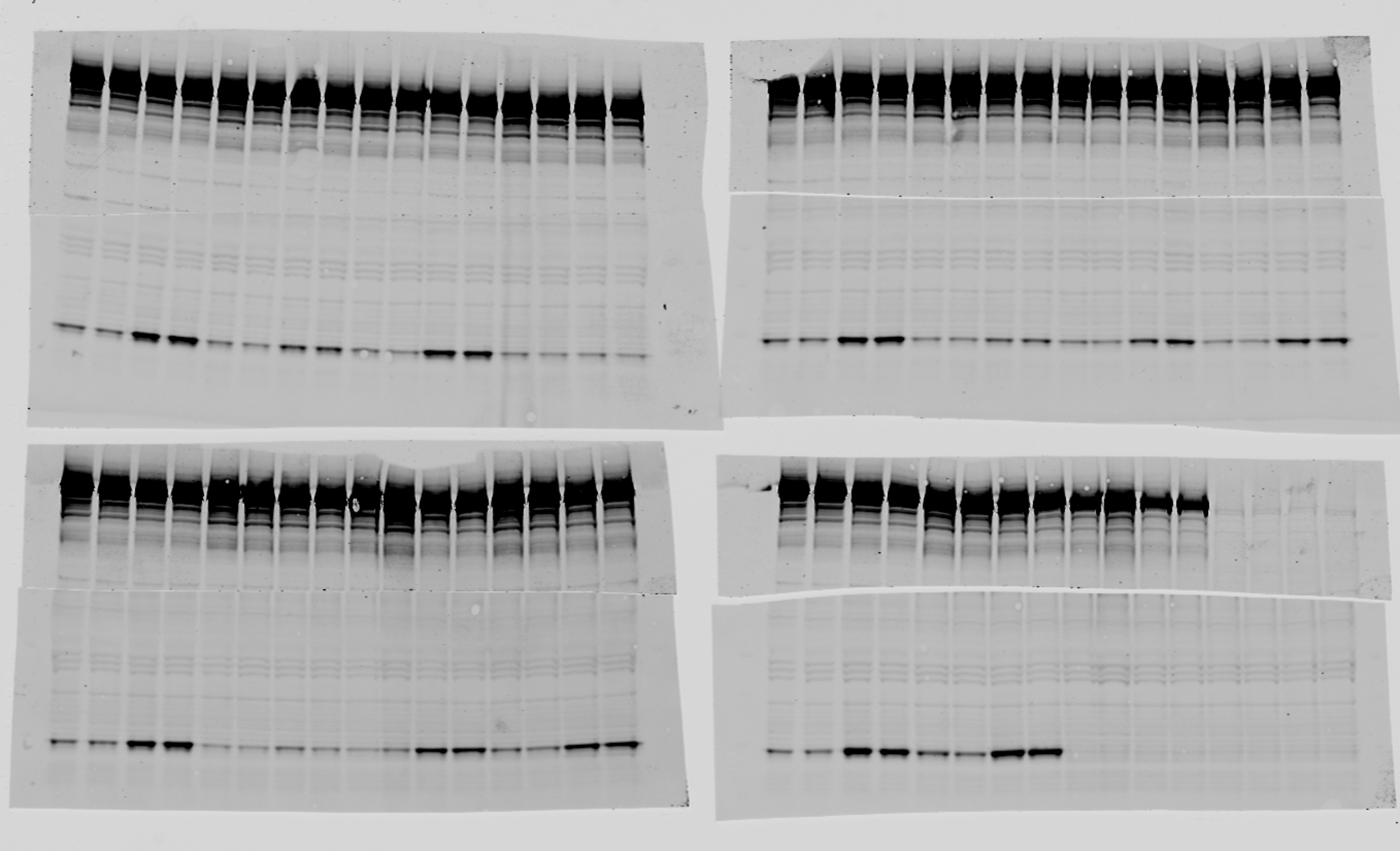

Supplement: Figure 3—figure supplement 4—source data 1. [file elife-79771-fig3-figsupp4-data1.zip › IB raw data/Fig.3.S4A_800(pRab10;pLRRK2)_High.tif]

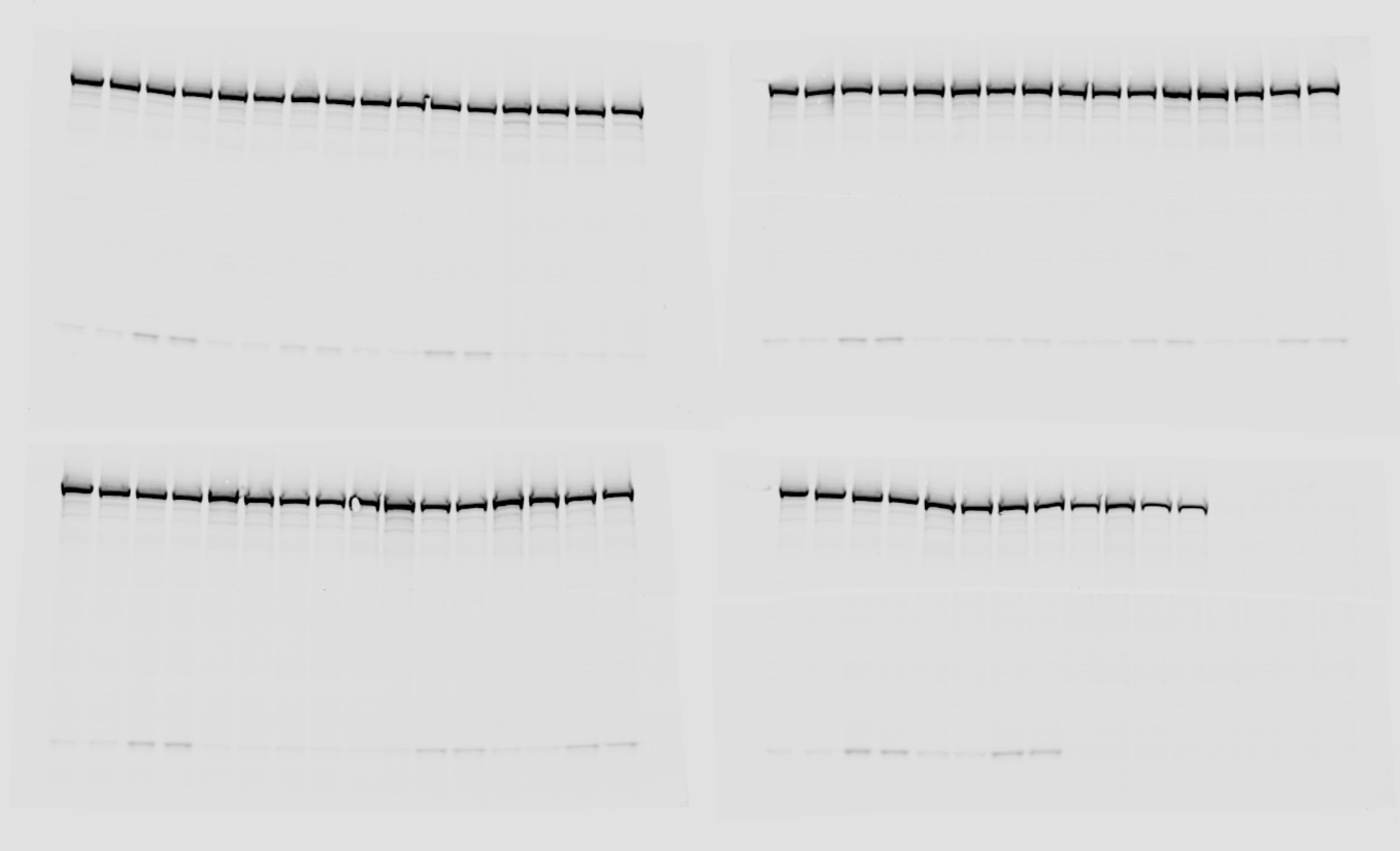

Supplement: Figure 3—figure supplement 4—source data 1. [file elife-79771-fig3-figsupp4-data1.zip › IB raw data/Fig.3.S4A_800(pRab10;pLRRK2)_Low.tif]

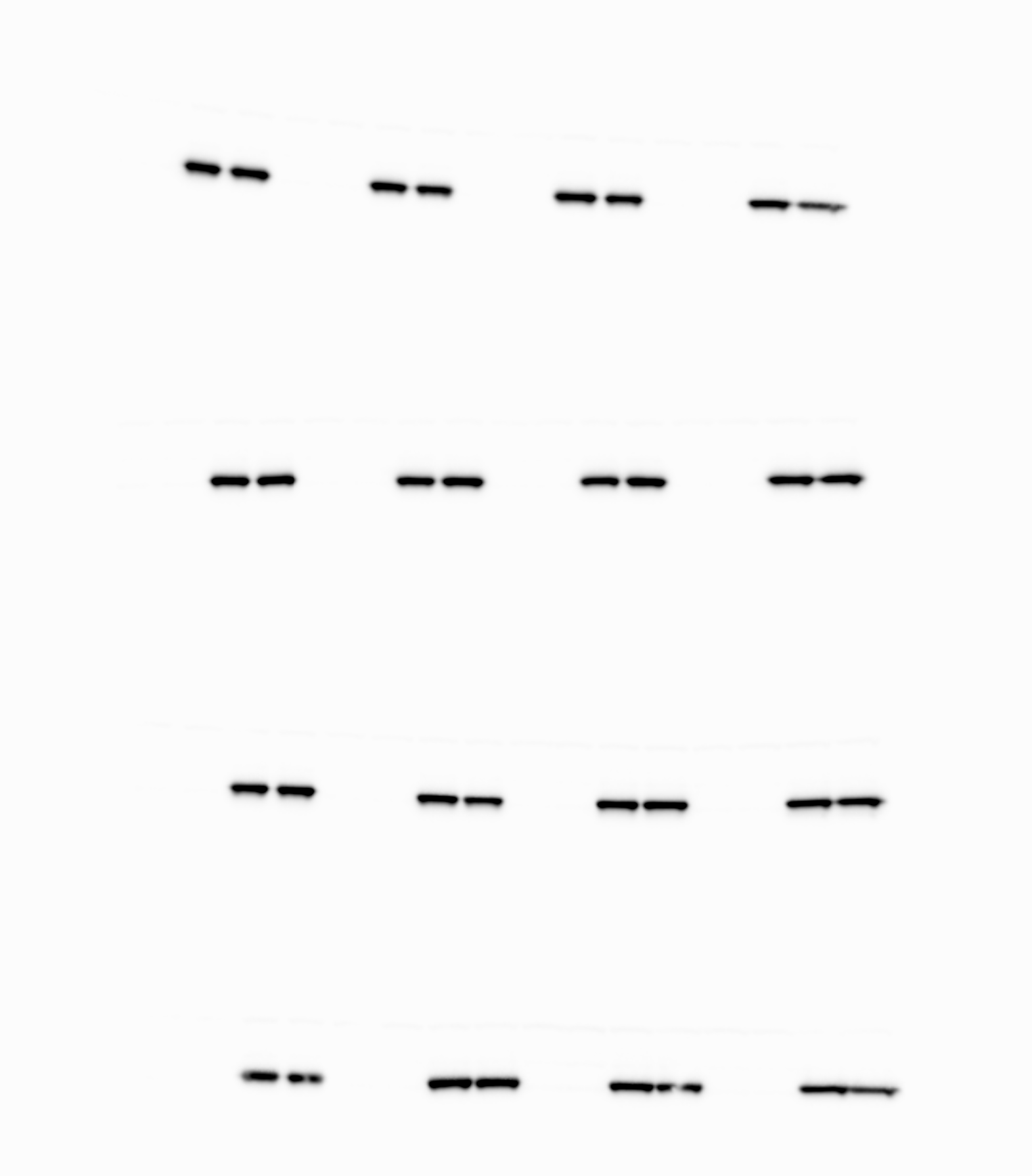

Supplement: Figure 3—figure supplement 4—source data 1. [file elife-79771-fig3-figsupp4-data1.zip › IB raw data/Fig.3.S4A_HA.tif]

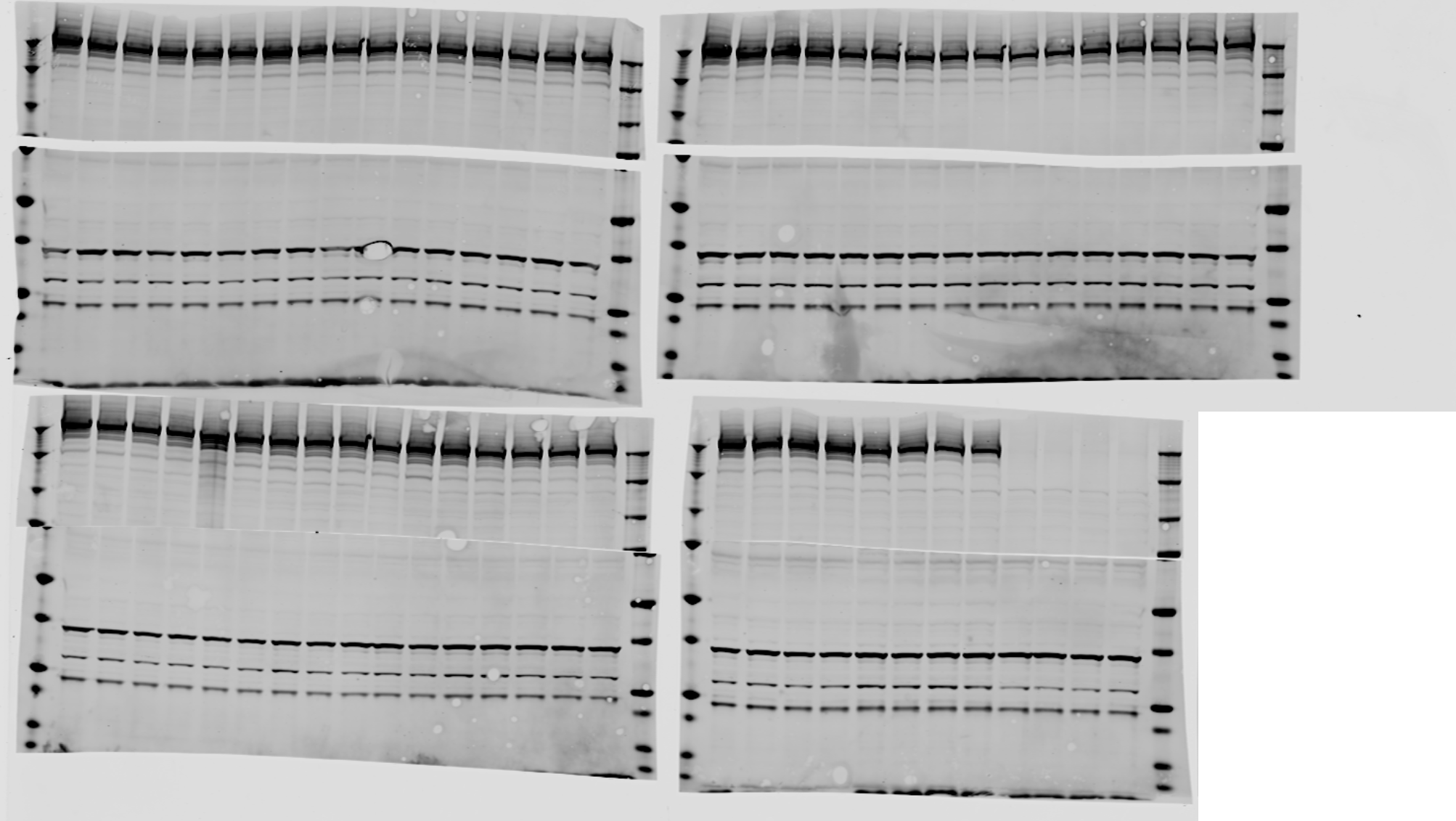

Supplement: Figure 3—figure supplement 4—source data 1. [file elife-79771-fig3-figsupp4-data1.zip › IB raw data/Fig.3.S4B_700(tRab10;tLRRK2)_High.tif]

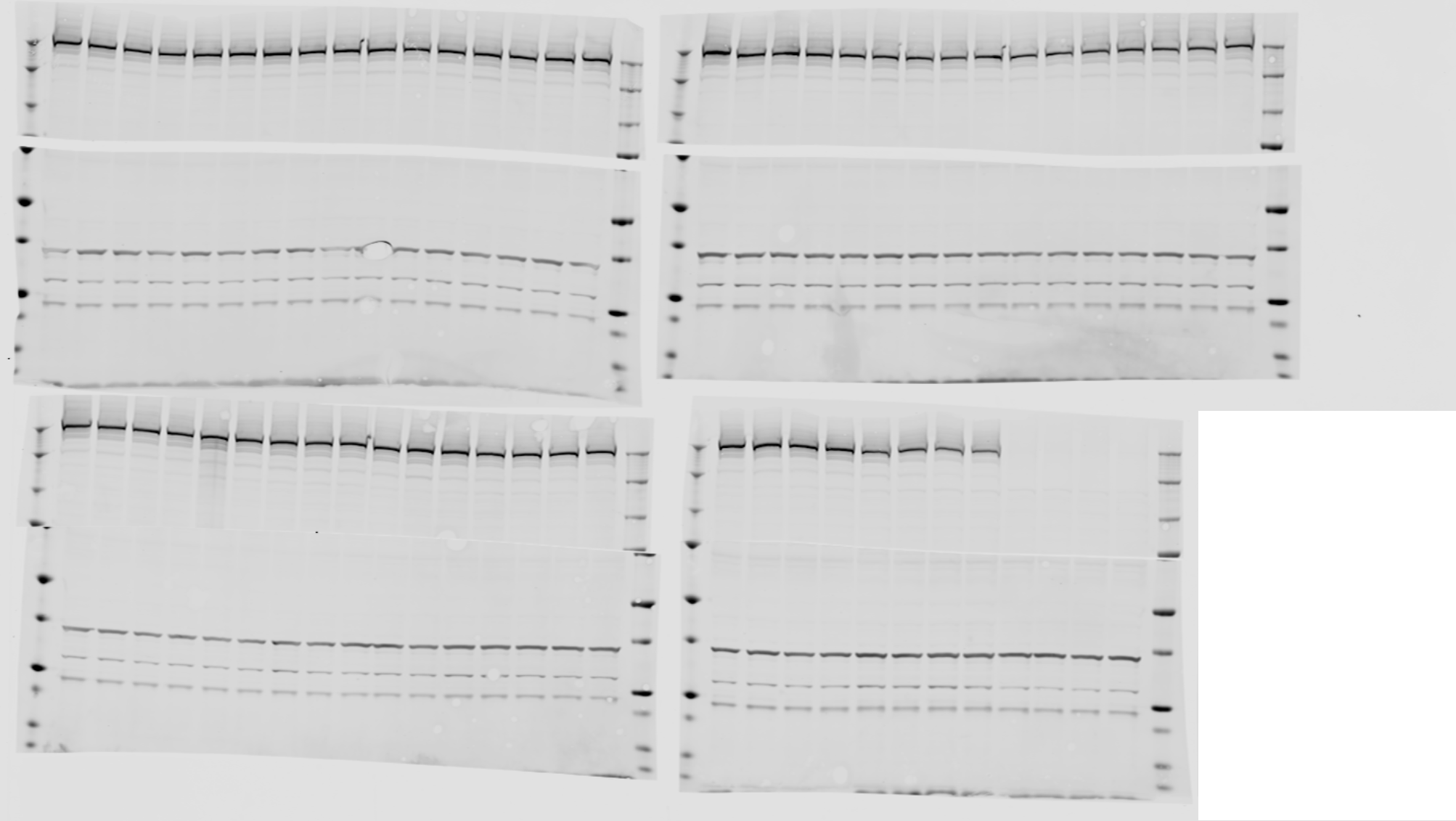

Supplement: Figure 3—figure supplement 4—source data 1. [file elife-79771-fig3-figsupp4-data1.zip › IB raw data/Fig.3.S4B_700(tRab10;tLRRK2)_Low.tif]

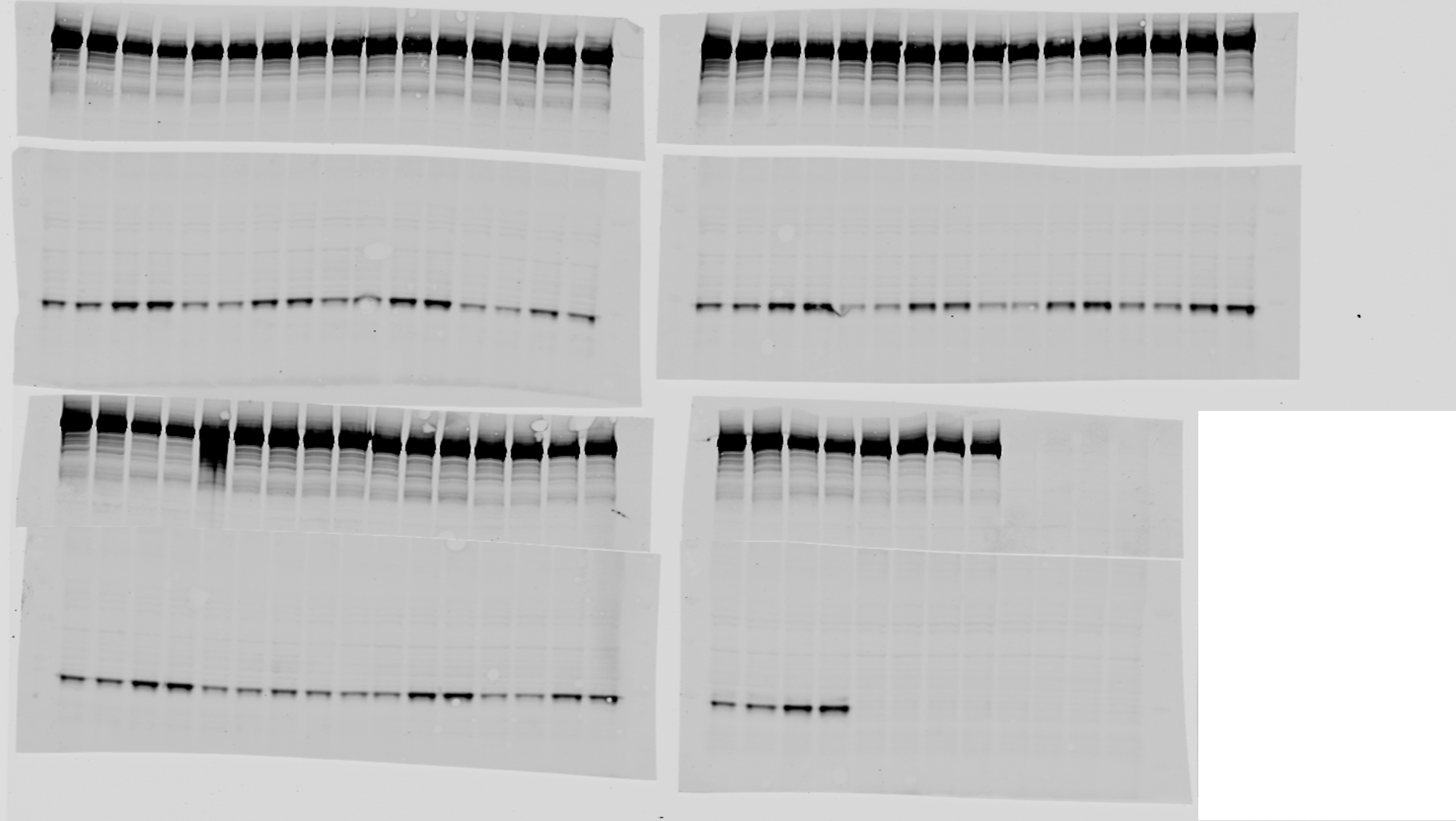

Supplement: Figure 3—figure supplement 4—source data 1. [file elife-79771-fig3-figsupp4-data1.zip › IB raw data/Fig.3.S4B_800(pRab10;pLRRK2)_High.tif]

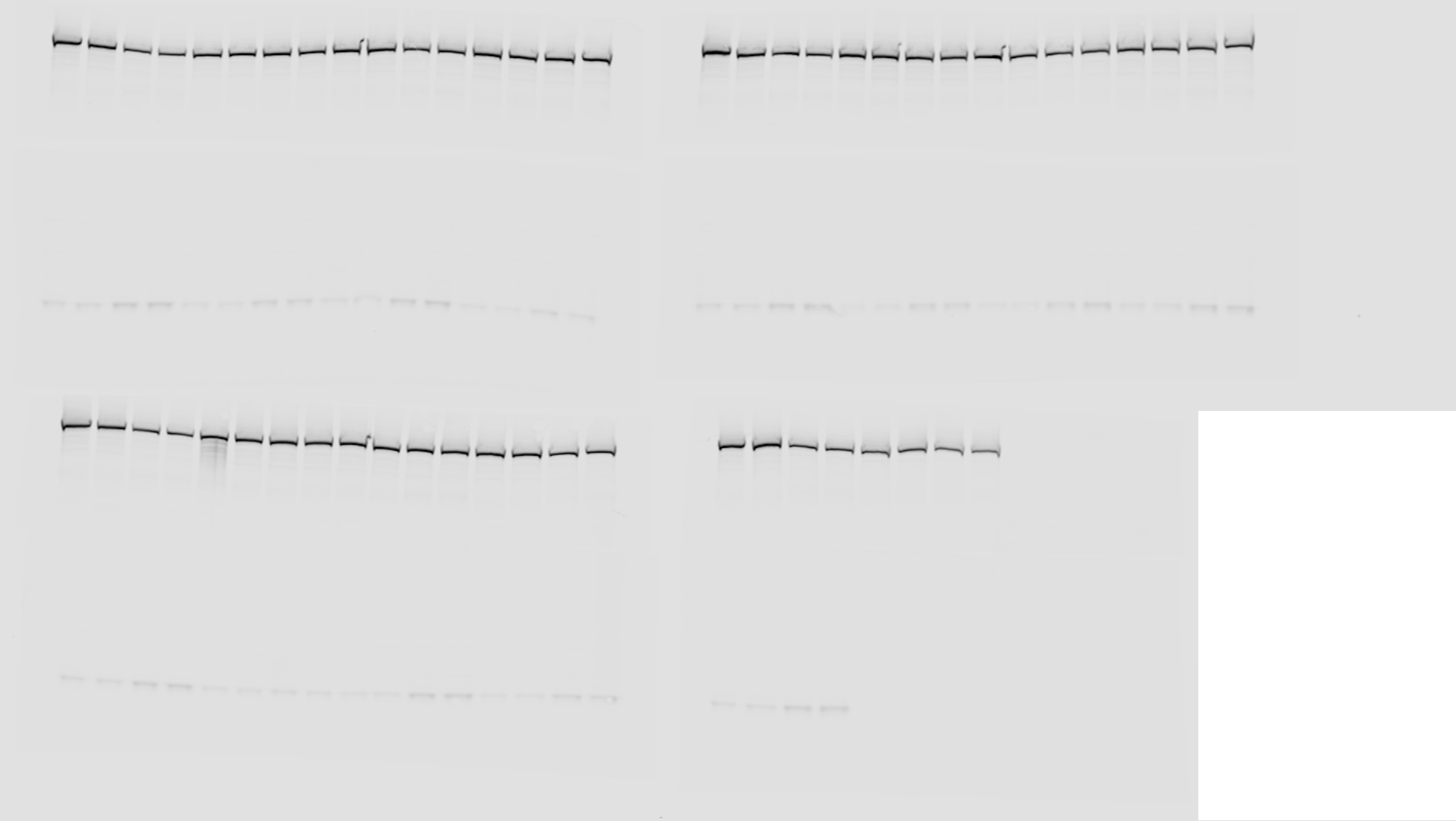

Supplement: Figure 3—figure supplement 4—source data 1. [file elife-79771-fig3-figsupp4-data1.zip › IB raw data/Fig.3.S4B_800(pRab10;pLRRK2)_Low.tif]

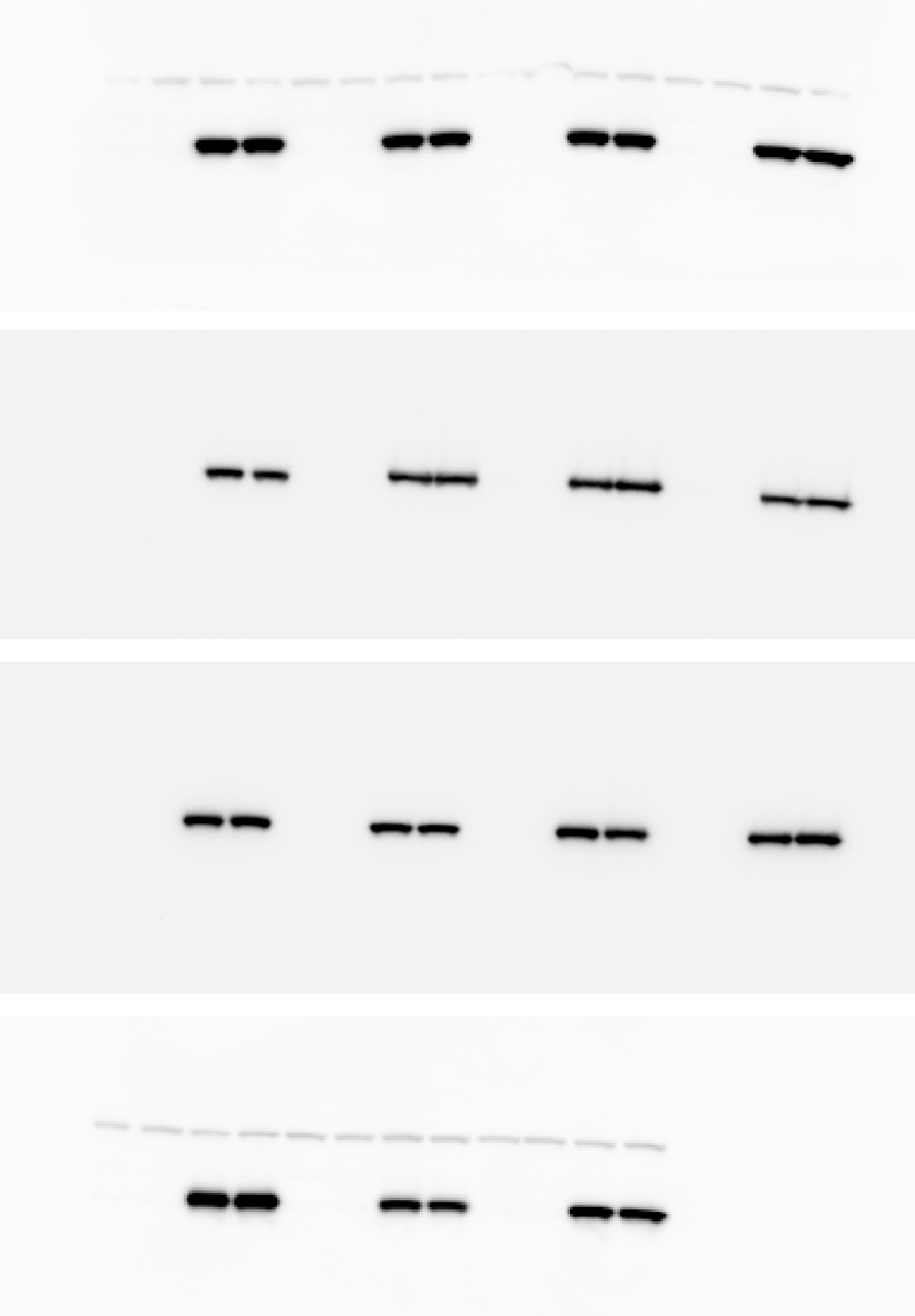

Supplement: Figure 3—figure supplement 4—source data 1. [file elife-79771-fig3-figsupp4-data1.zip › IB raw data/Fig.3.S4B_HA.tif]

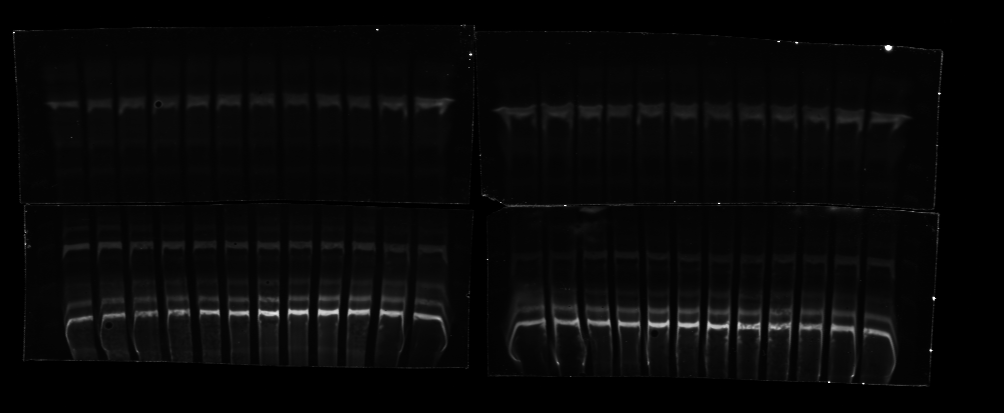

Supplement: Figure 7—source data 1. [file elife-79771-fig7-data1.zip › Figure7RAWdata/Figure7C_D_source_data_3_800.TIF]

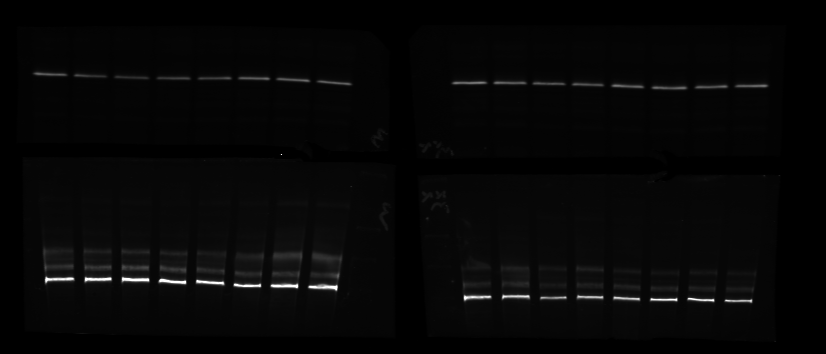

Supplement: Figure 7—source data 1. [file elife-79771-fig7-data1.zip › Figure7RAWdata/Figure7C_D_source_data_2_800.TIF]

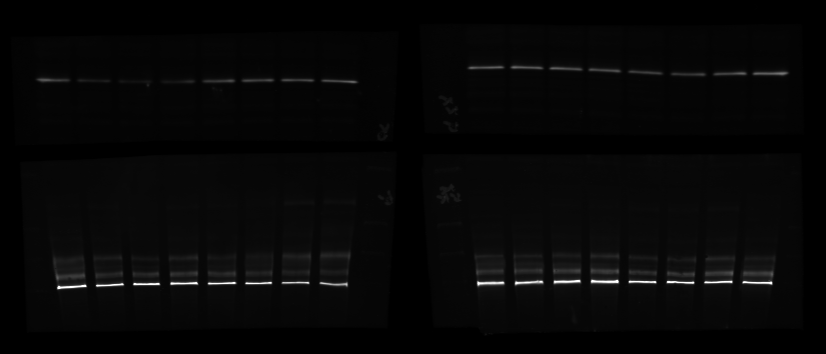

Supplement: Figure 7—source data 1. [file elife-79771-fig7-data1.zip › Figure7RAWdata/Figure7A_B_source_data_3_800.TIF]

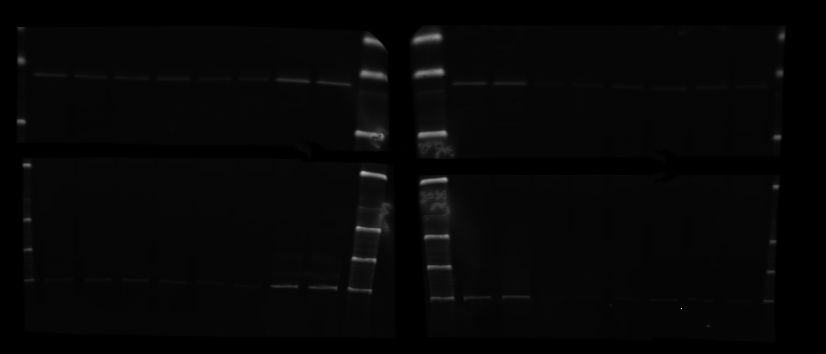

Supplement: Figure 7—source data 1. [file elife-79771-fig7-data1.zip › Figure7RAWdata/Figure7C_D_source_data_2_700.TIF]

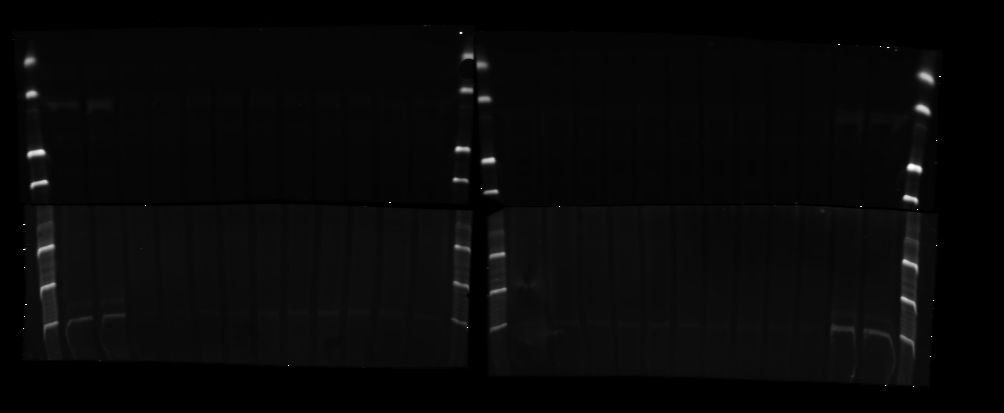

Supplement: Figure 7—source data 1. [file elife-79771-fig7-data1.zip › Figure7RAWdata/Figure7C_D_source_data_3_700.TIF]

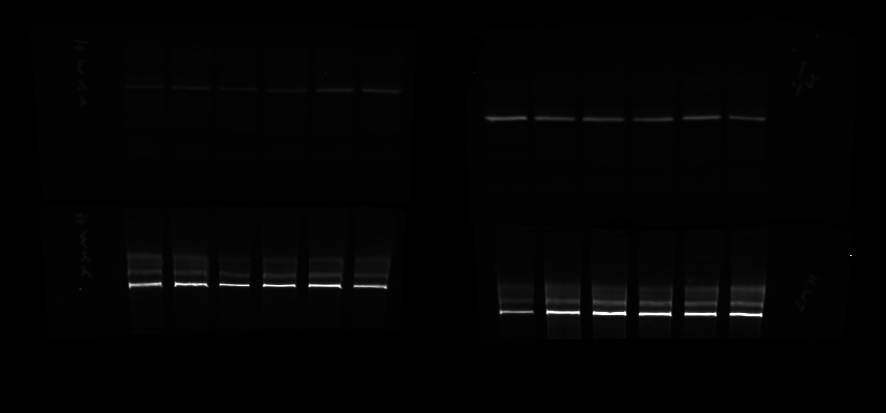

Supplement: Figure 7—source data 1. [file elife-79771-fig7-data1.zip › Figure7RAWdata/Figure7C_D_source_data_1_800.TIF]

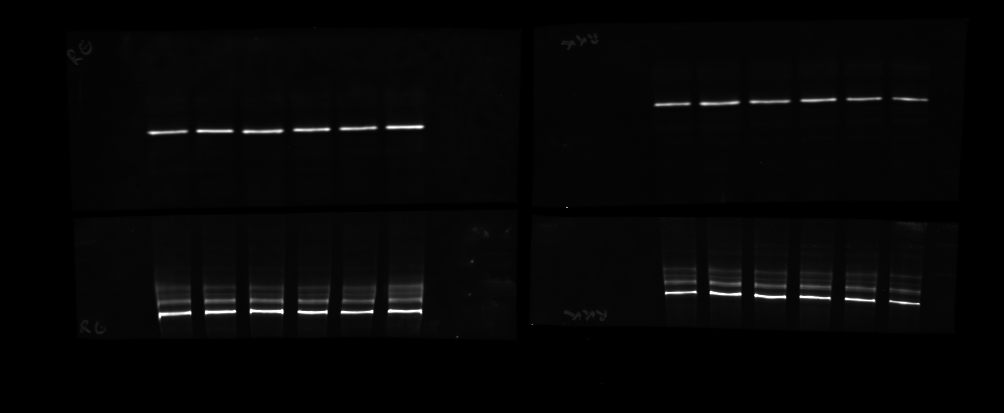

Supplement: Figure 7—source data 1. [file elife-79771-fig7-data1.zip › Figure7RAWdata/Figure7A_B_source_data_2_800.TIF]

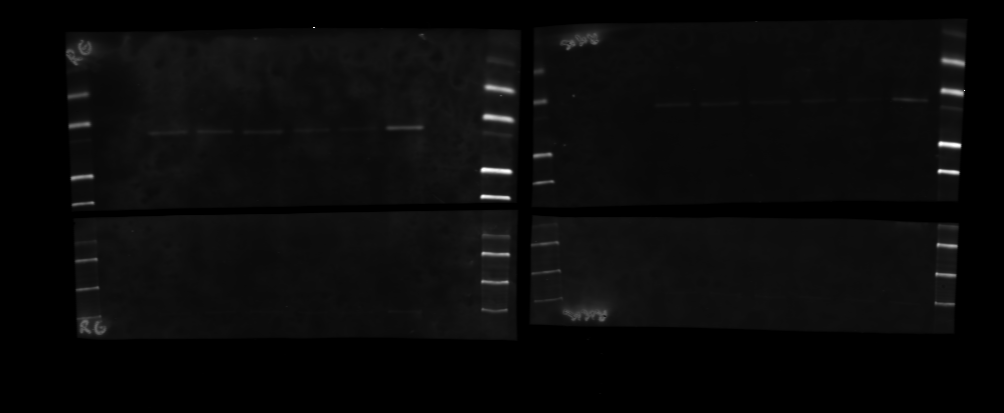

Supplement: Figure 7—source data 1. [file elife-79771-fig7-data1.zip › Figure7RAWdata/Figure7A_B_source_data_2_700.TIF]

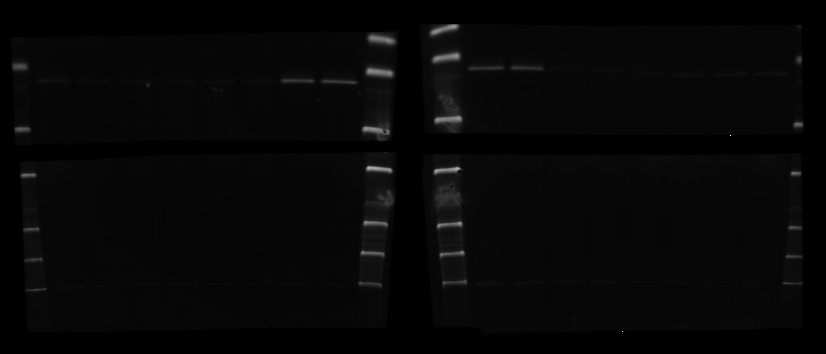

Supplement: Figure 7—source data 1. [file elife-79771-fig7-data1.zip › Figure7RAWdata/Figure7A_B_source_data_3_700.TIF]

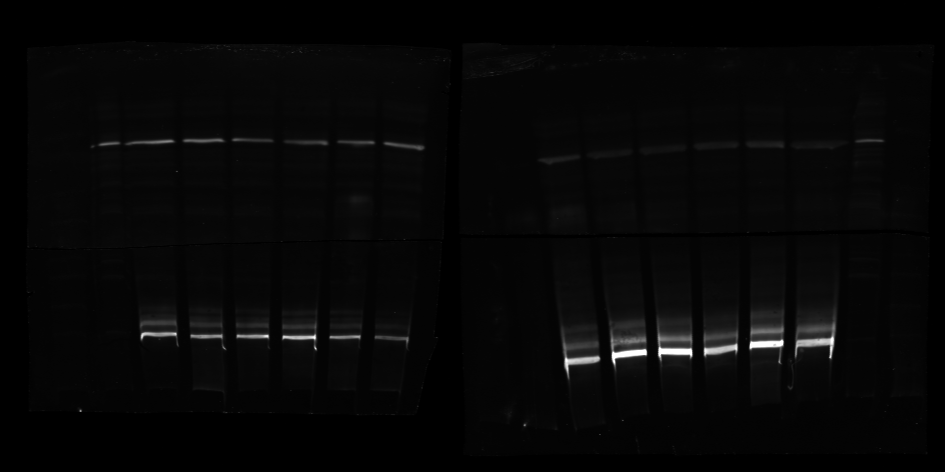

Supplement: Figure 7—source data 1. [file elife-79771-fig7-data1.zip › Figure7RAWdata/Figure7A_B_source_data_1_800.TIF]

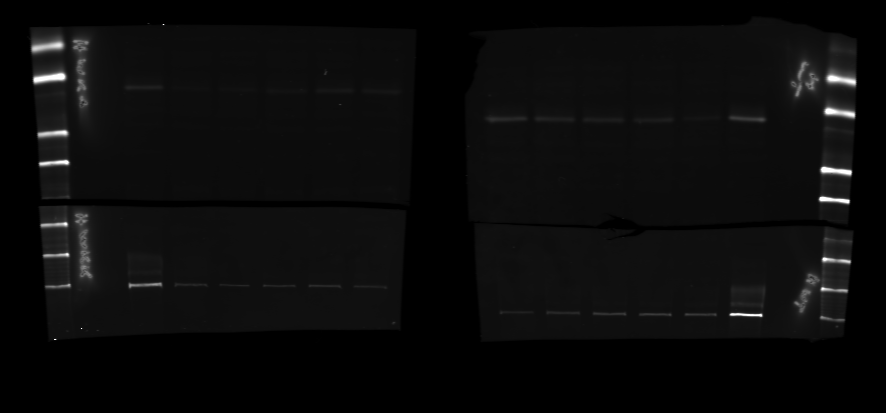

Supplement: Figure 7—source data 1. [file elife-79771-fig7-data1.zip › Figure7RAWdata/Figure7C_D_source_data_1_700.TIF]

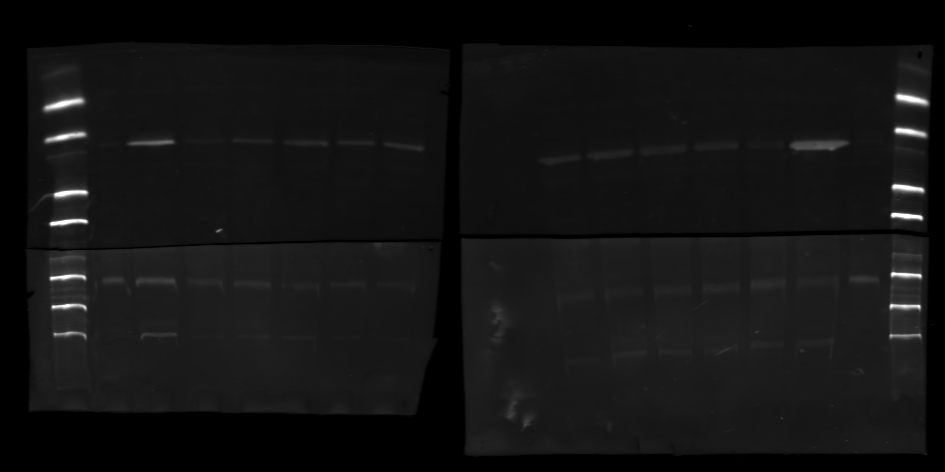

Supplement: Figure 7—source data 1. [file elife-79771-fig7-data1.zip › Figure7RAWdata/Figure7A_B_source_data_1_700.TIF]

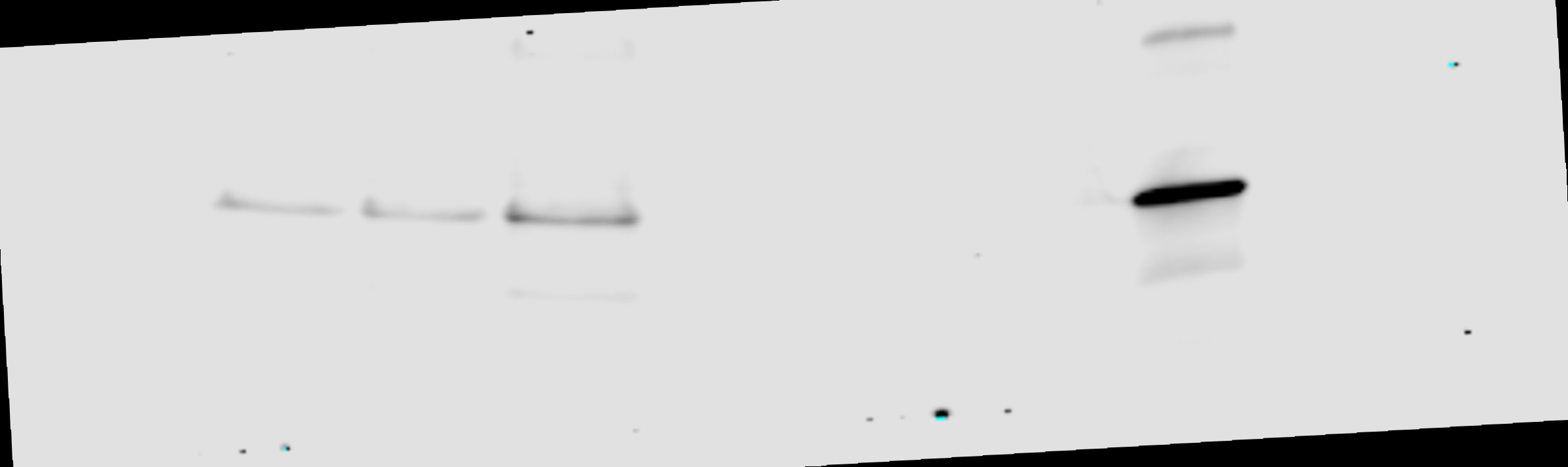

Supplement: Figure 8—figure supplement 2—source data 1. [file elife-79771-fig8-figsupp2-data1.zip › Figure 8S2/rbxpRab10.tif]

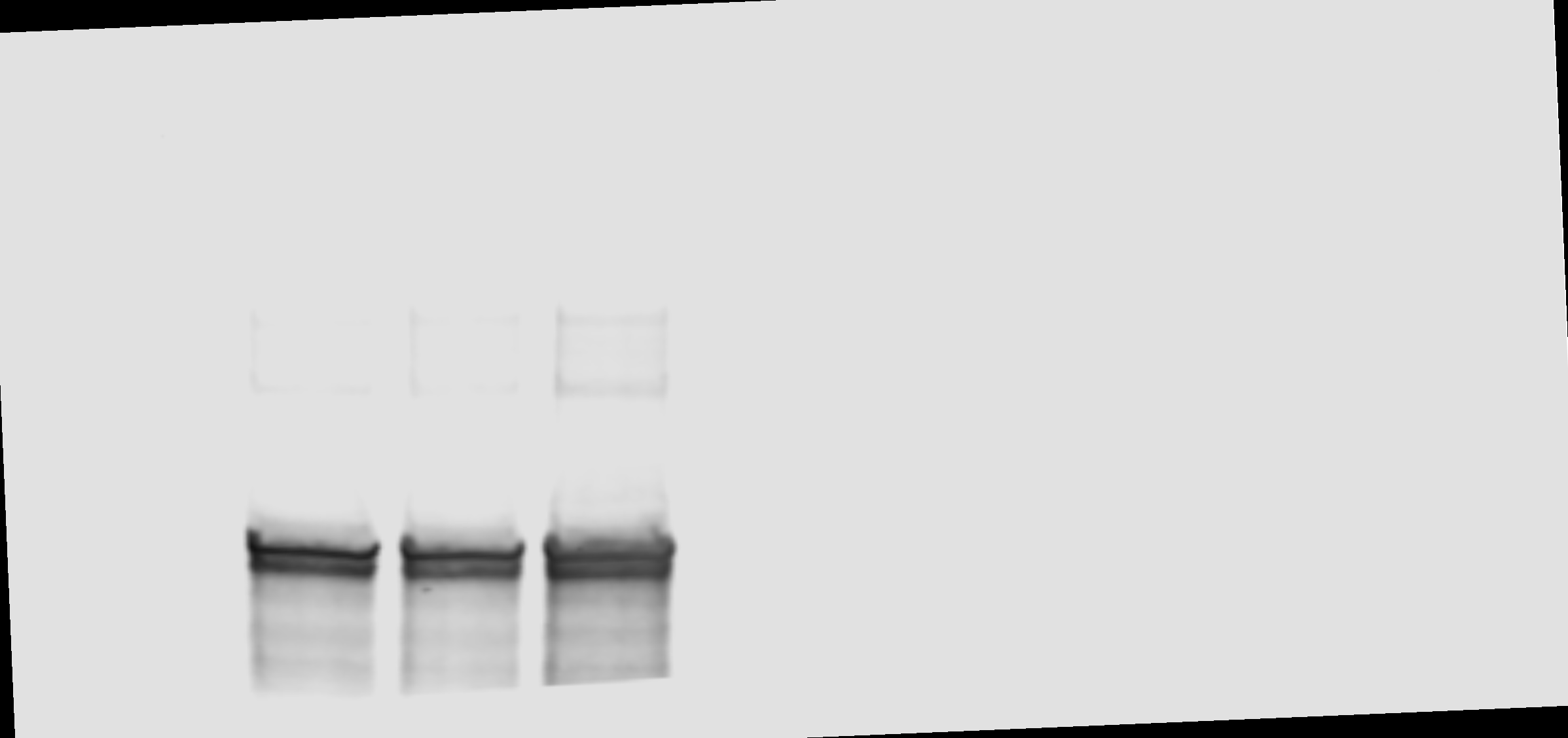

Supplement: Figure 8—figure supplement 2—source data 1. [file elife-79771-fig8-figsupp2-data1.zip › Figure 8S2/rbxLRRK2.tif]

Figure 8 - Supplement 2

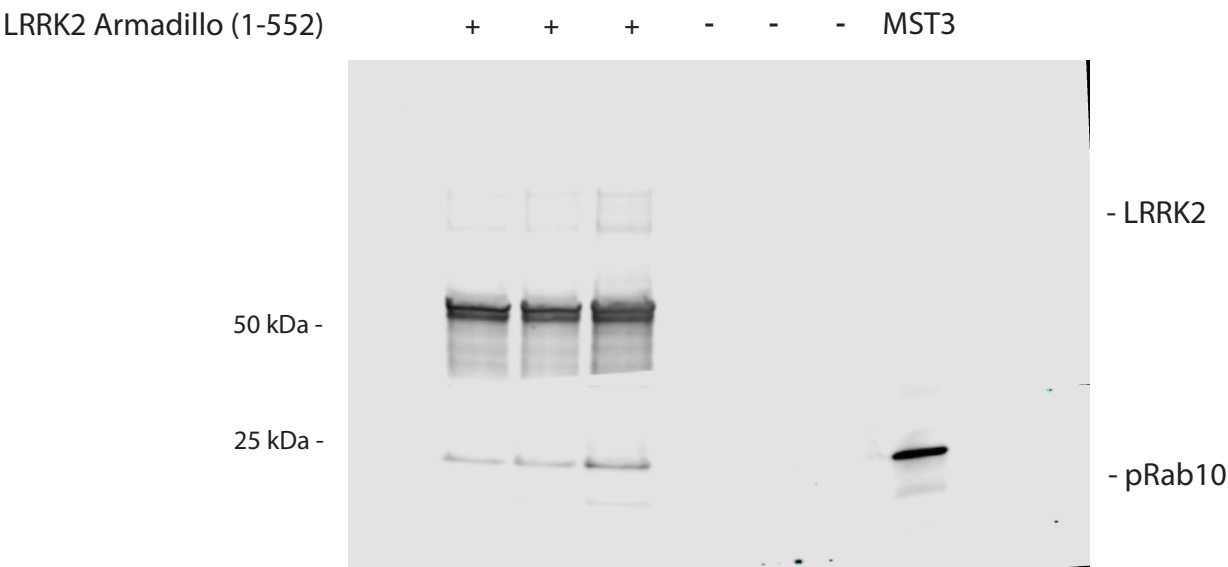

Supplement: Figure 8—figure supplement 2—source data 2. [file elife-79771-fig8-figsupp2-data2.pdf]

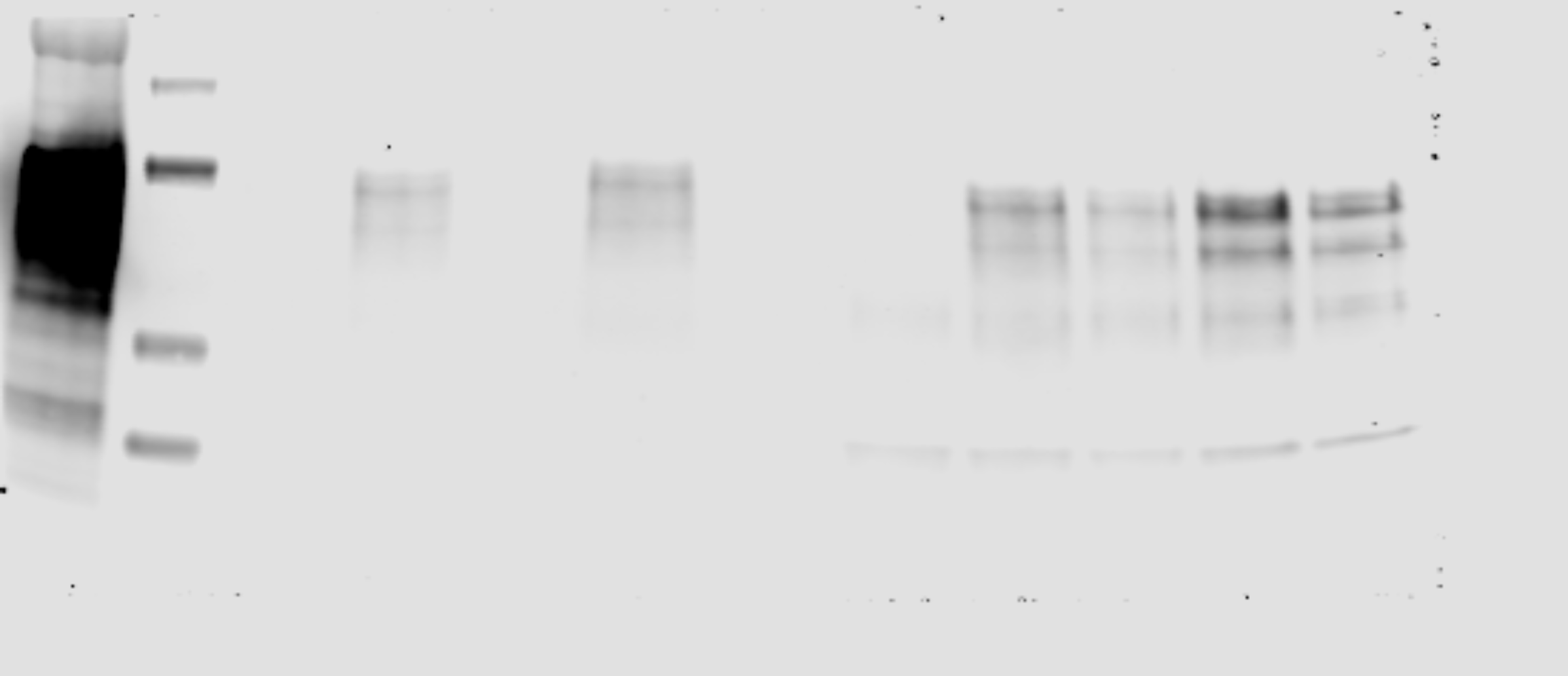

Supplement: Figure 9—source data 1. [file elife-79771-fig9-data1.zip › Figure 9A/rbxpRab10 4.tif]

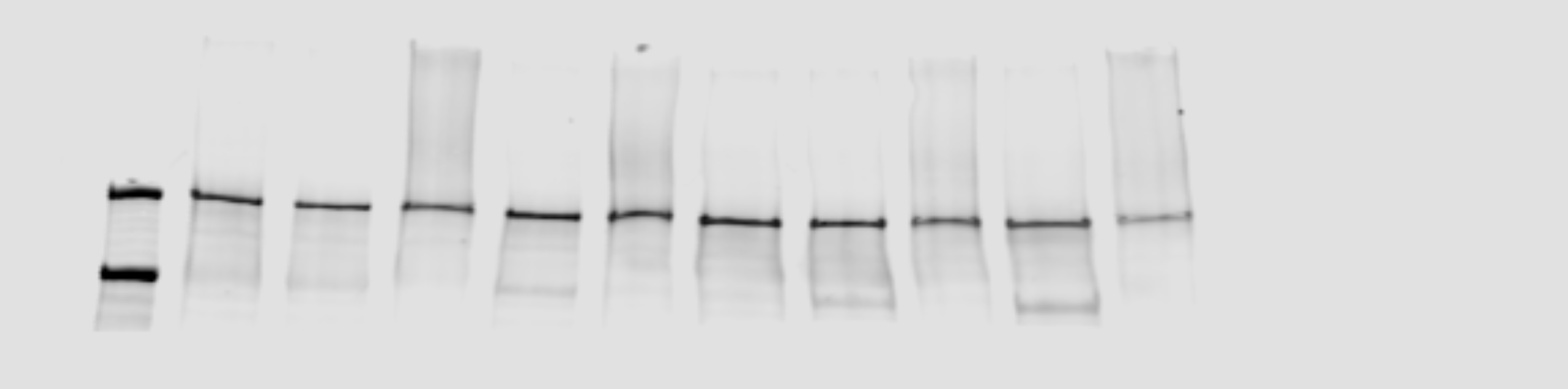

Supplement: Figure 9—source data 1. [file elife-79771-fig9-data1.zip › Figure 9A/msxLRRK2 3.tif]

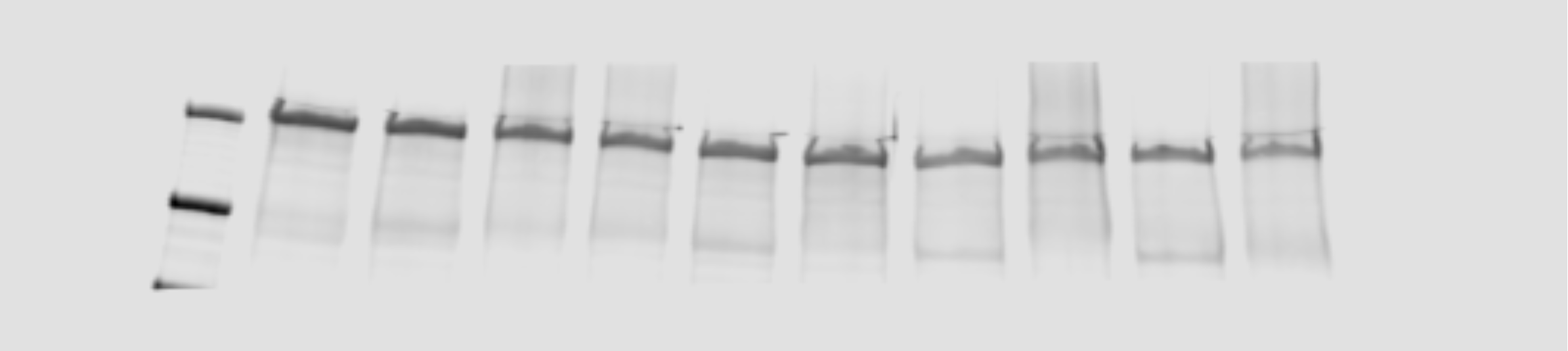

Supplement: Figure 9—source data 1. [file elife-79771-fig9-data1.zip › Figure 9A/msxLRRK2 2.tif]

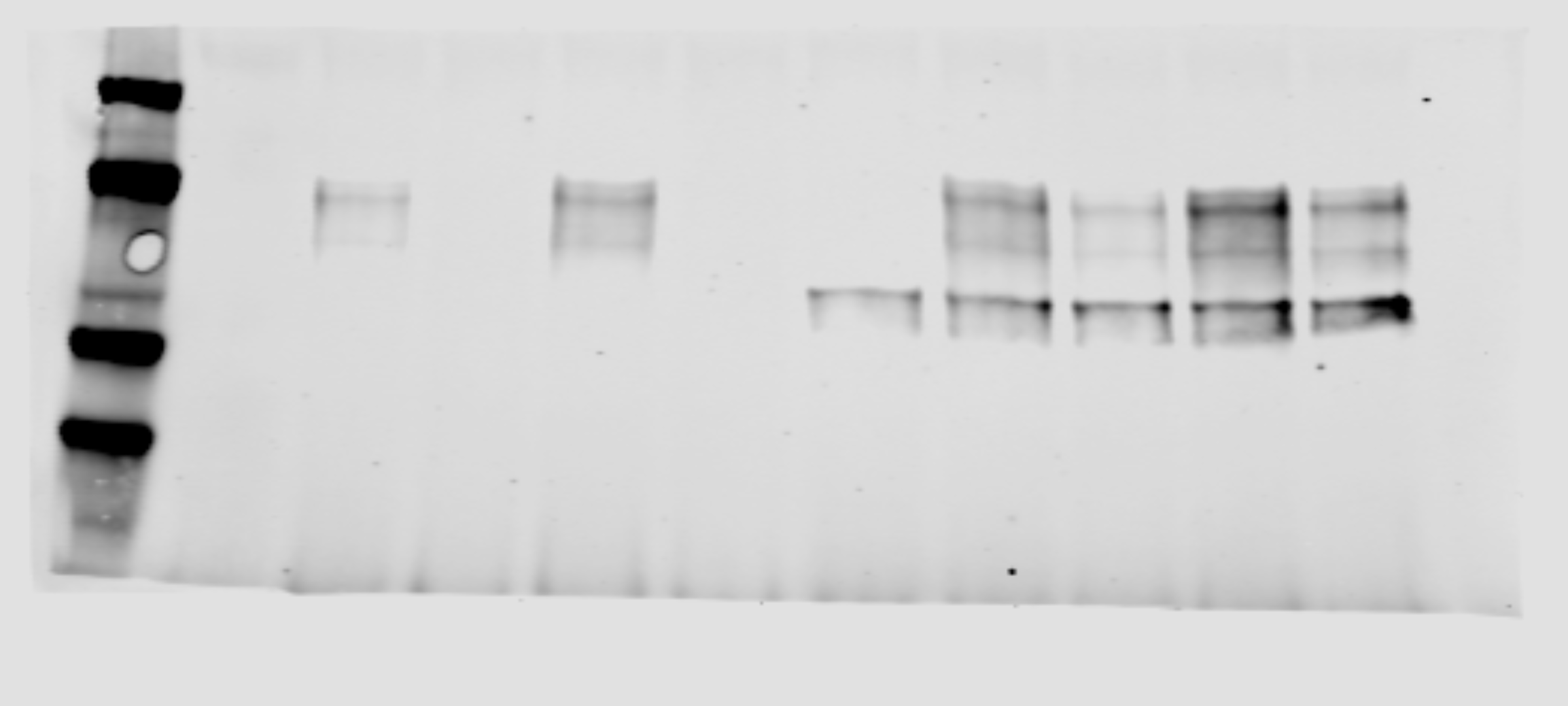

Supplement: Figure 9—source data 1. [file elife-79771-fig9-data1.zip › Figure 9A/rbxpRab10 5.tif]

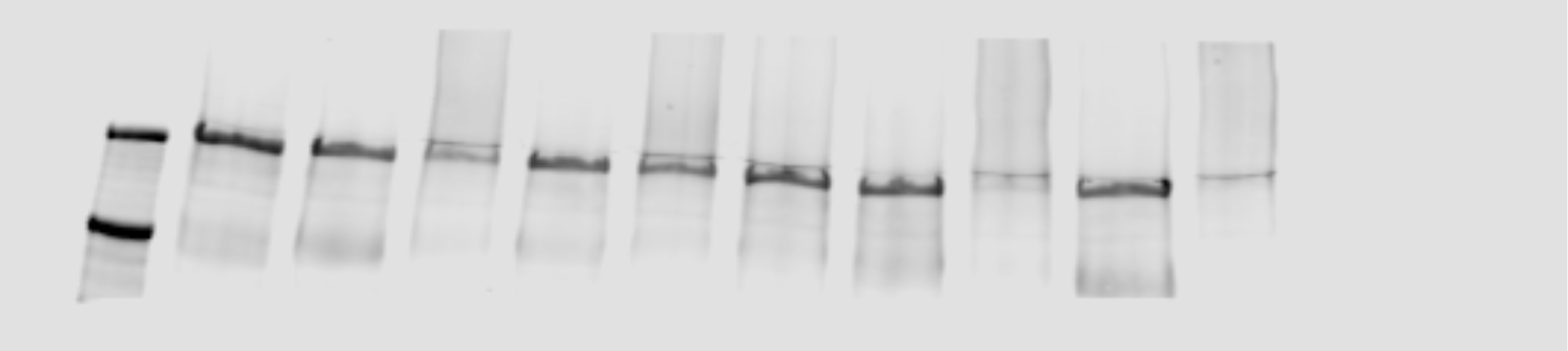

Supplement: Figure 9—source data 1. [file elife-79771-fig9-data1.zip › Figure 9A/msxLRRK2 1.tif]

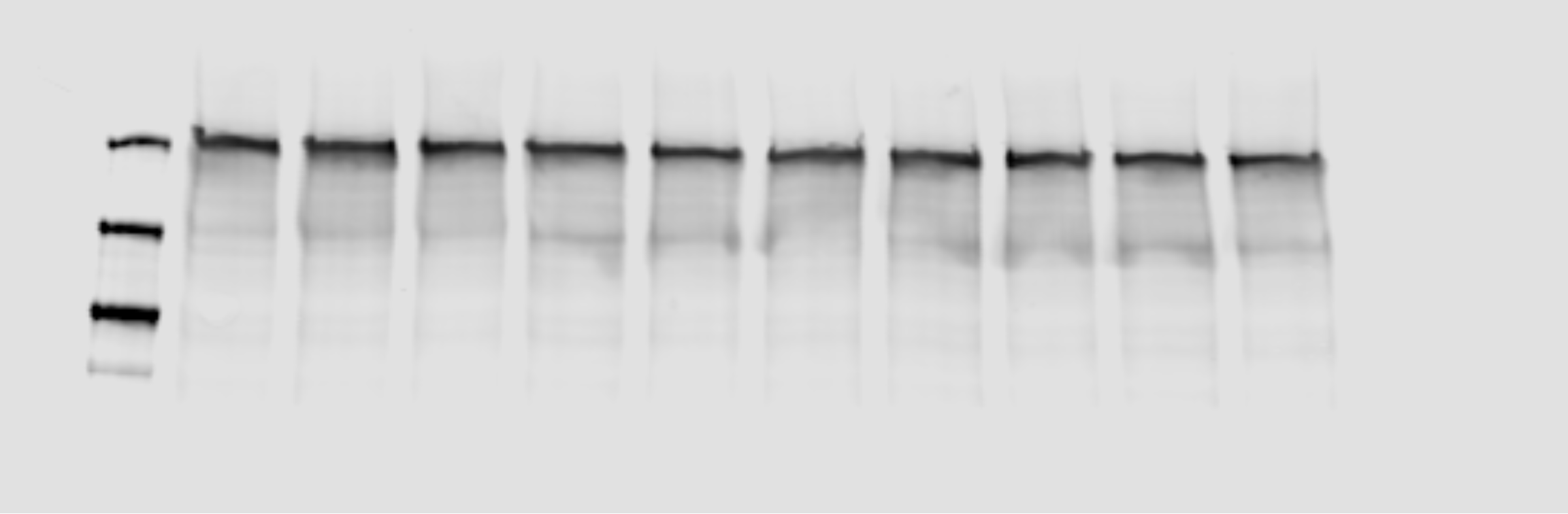

Supplement: Figure 9—source data 1. [file elife-79771-fig9-data1.zip › Figure 9A/msxLRRK2 5.tif]

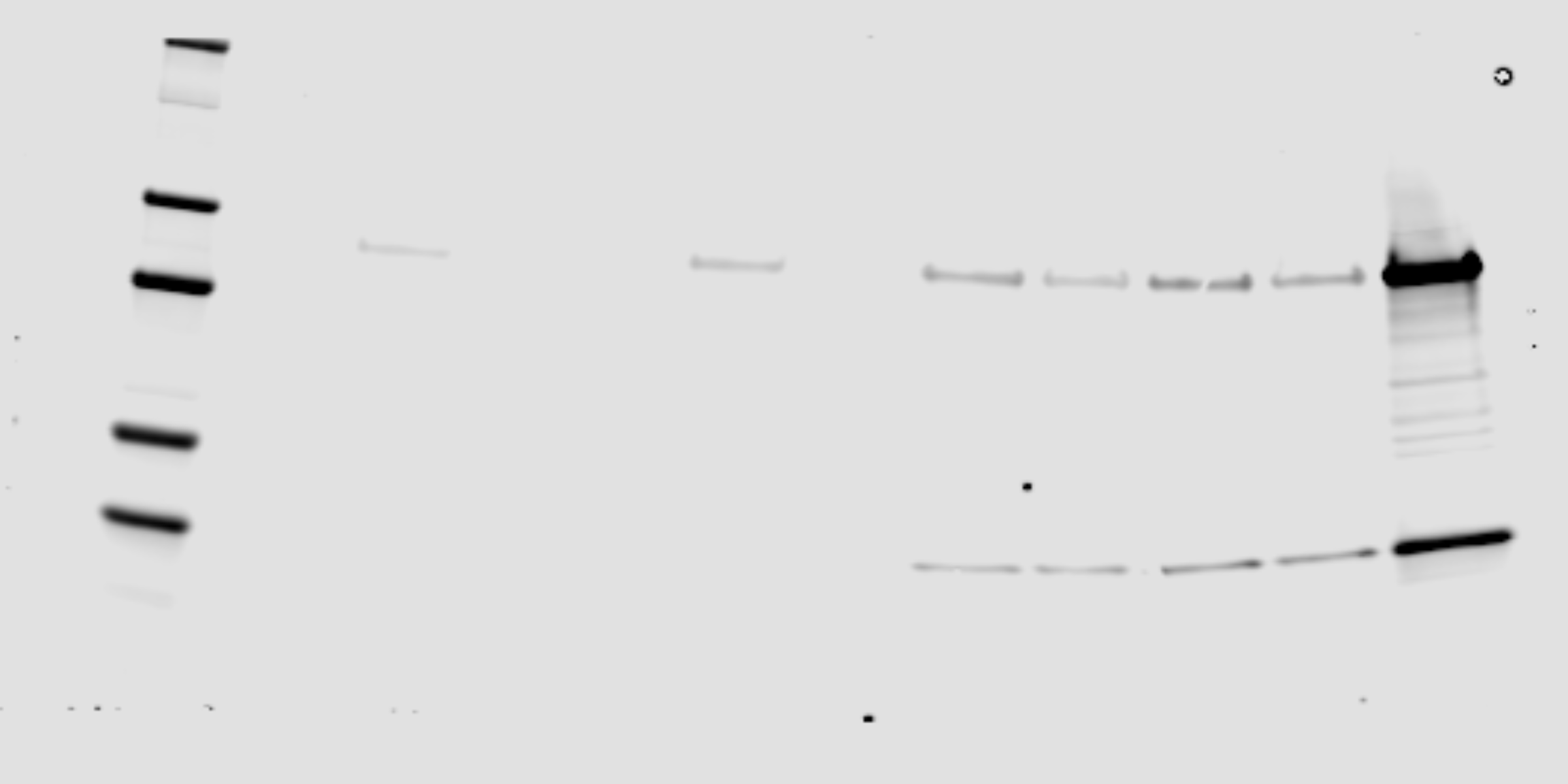

Supplement: Figure 9—source data 1. [file elife-79771-fig9-data1.zip › Figure 9A/rbxpRab10 2.tif]

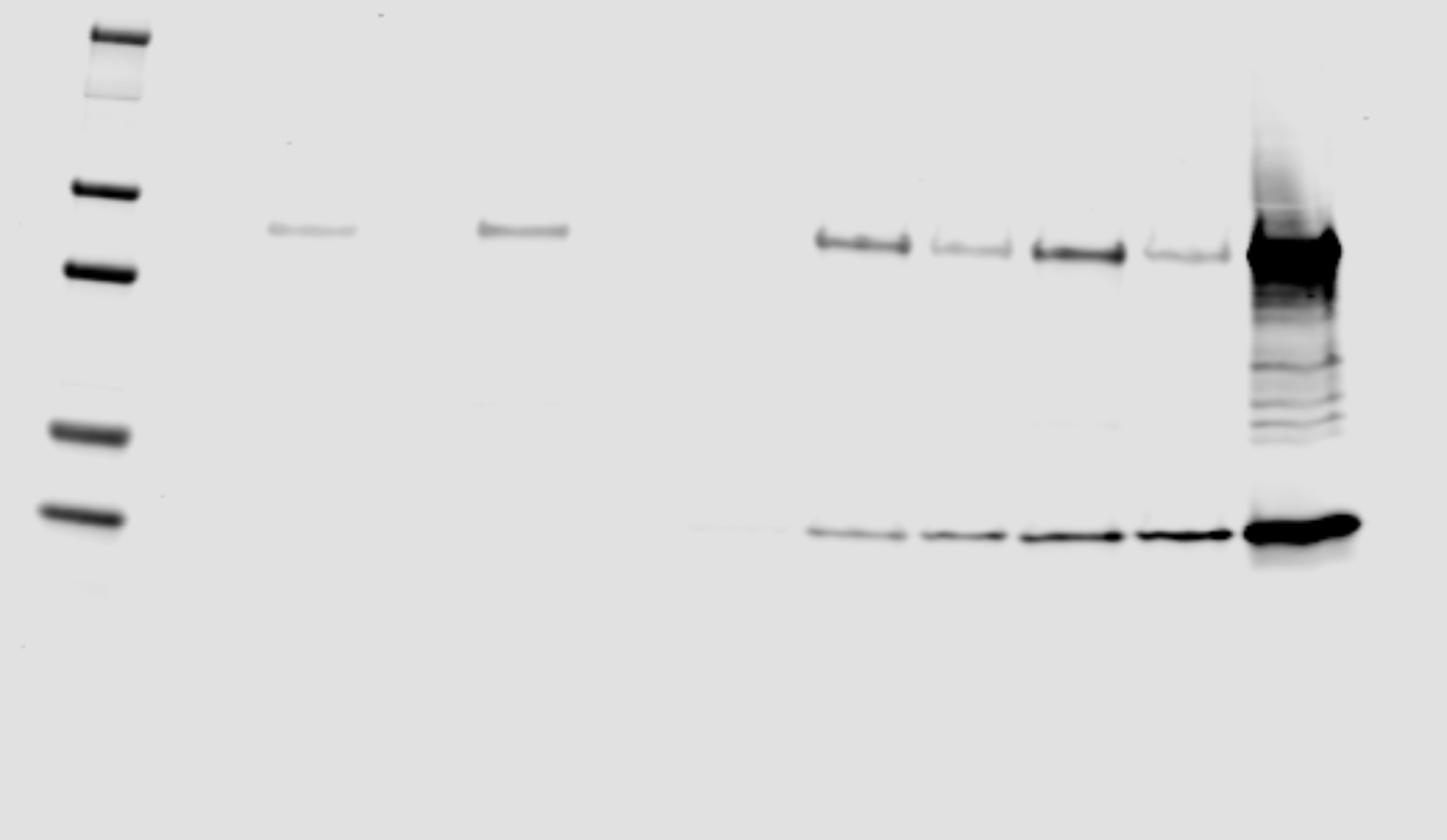

Supplement: Figure 9—source data 1. [file elife-79771-fig9-data1.zip › Figure 9A/rbxpRab10 3.tif]

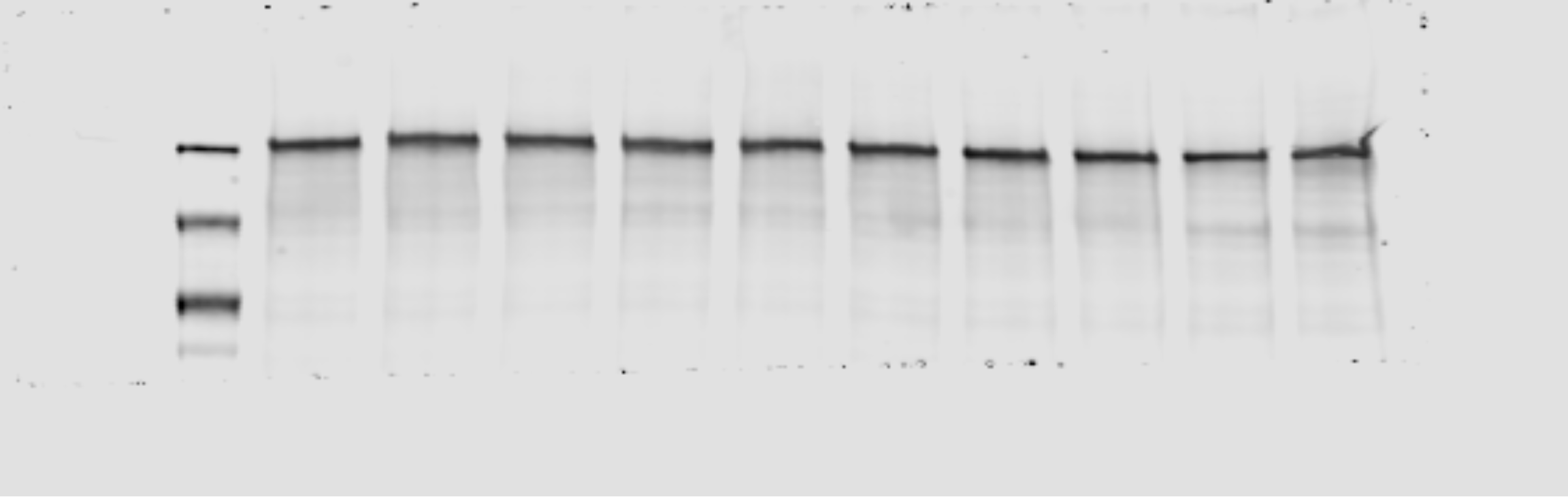

Supplement: Figure 9—source data 1. [file elife-79771-fig9-data1.zip › Figure 9A/msxLRRK2 4.tif]

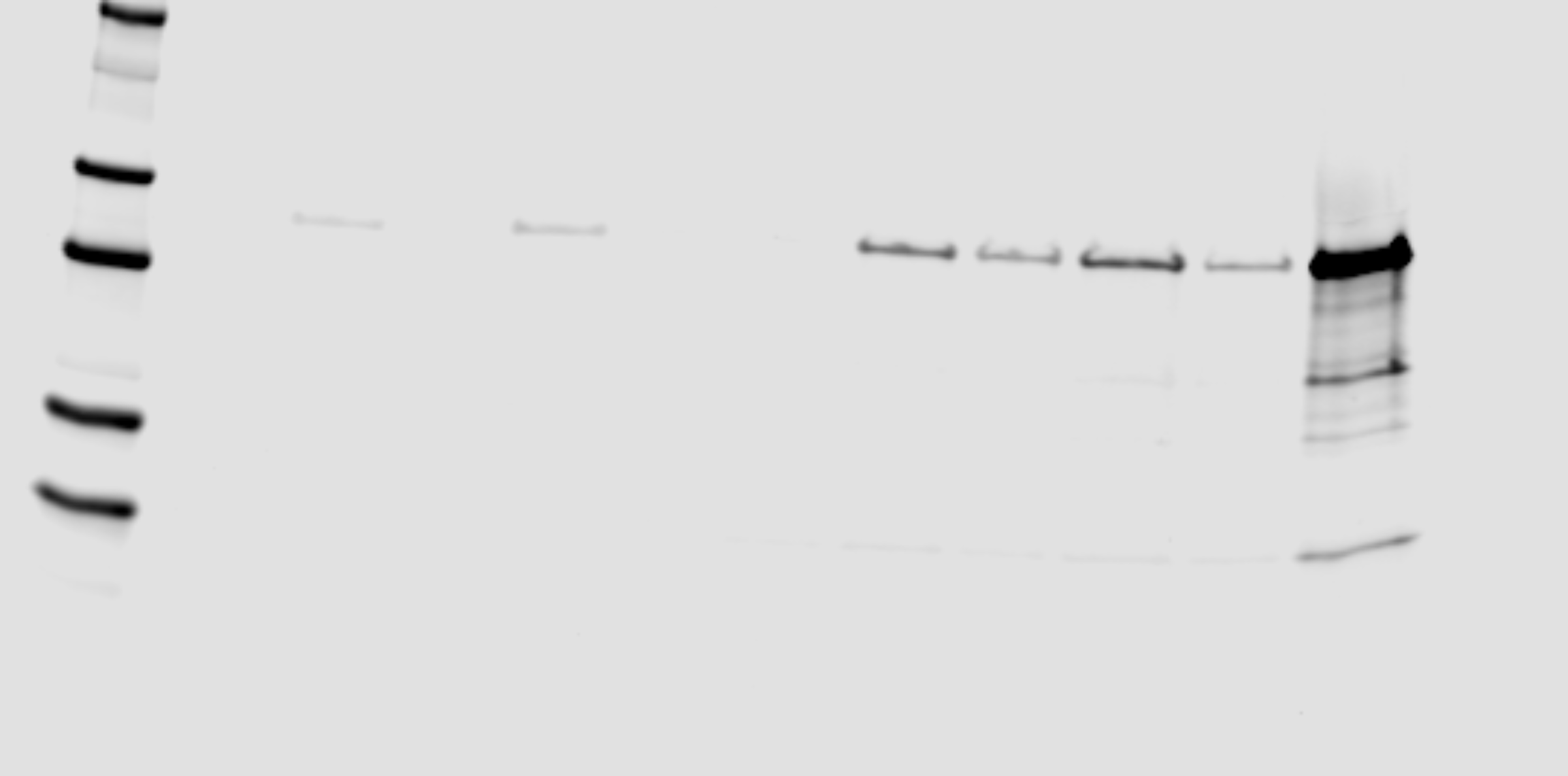

Supplement: Figure 9—source data 1. [file elife-79771-fig9-data1.zip › Figure 9A/rbxpRab10 1.tif]

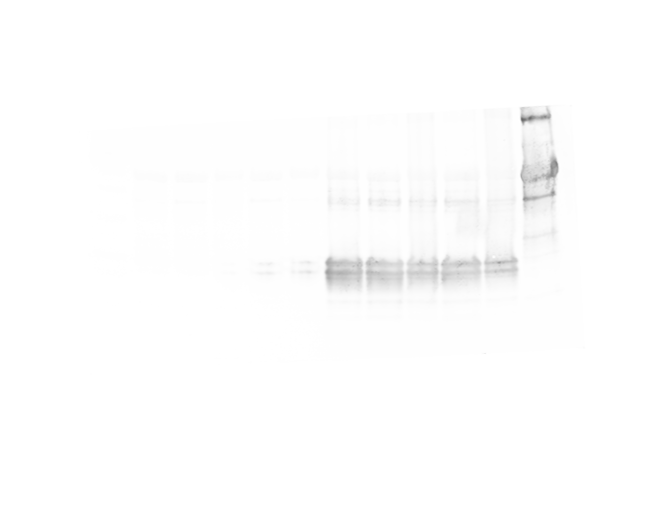

Supplement: Figure 9—source data 1. [file elife-79771-fig9-data1.zip › Figure 9A/rbxpRab8 1.TIF]

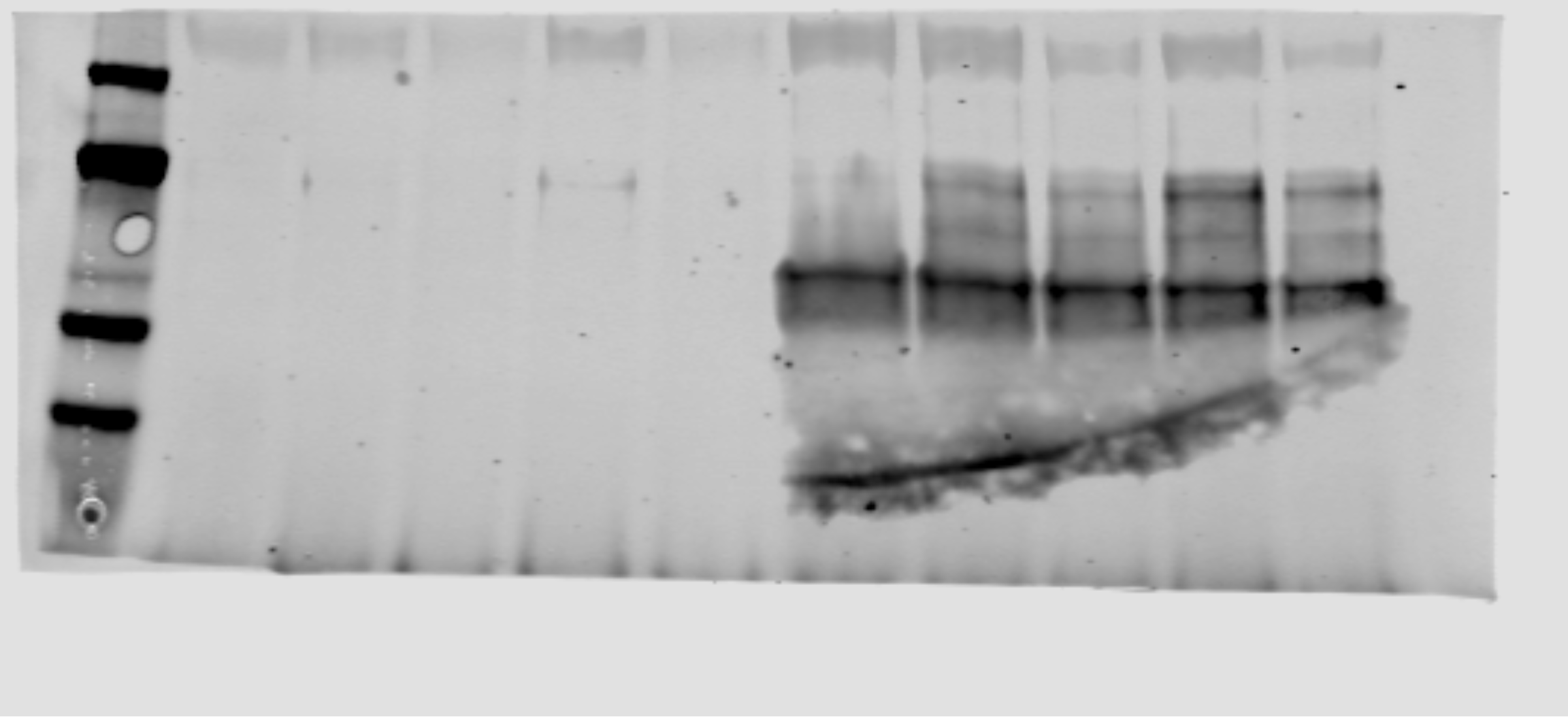

Supplement: Figure 9—source data 1. [file elife-79771-fig9-data1.zip › Figure 9A/rbxpRab8 5.tif]

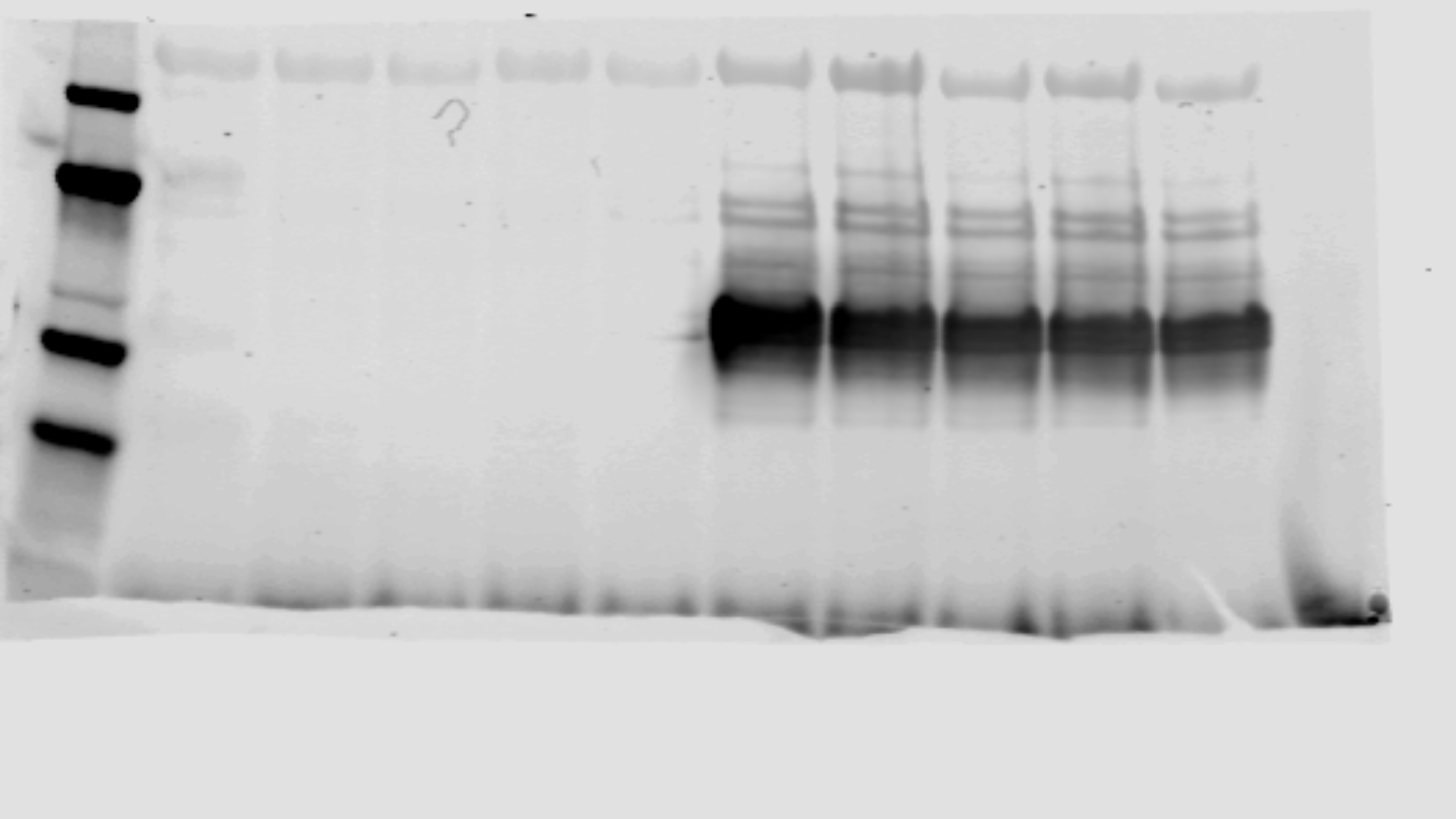

Supplement: Figure 9—source data 1. [file elife-79771-fig9-data1.zip › Figure 9B/rbxRab8.tif]

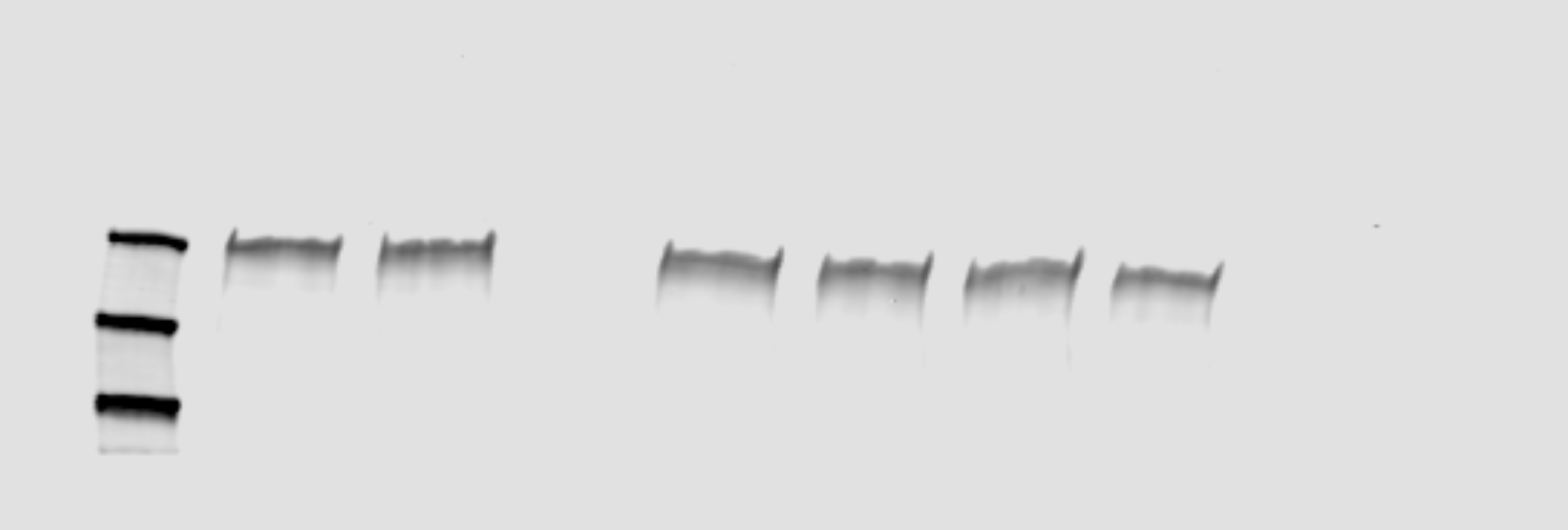

Supplement: Figure 9—source data 1. [file elife-79771-fig9-data1.zip › Figure 9B/msxLRRK2 2.tif]

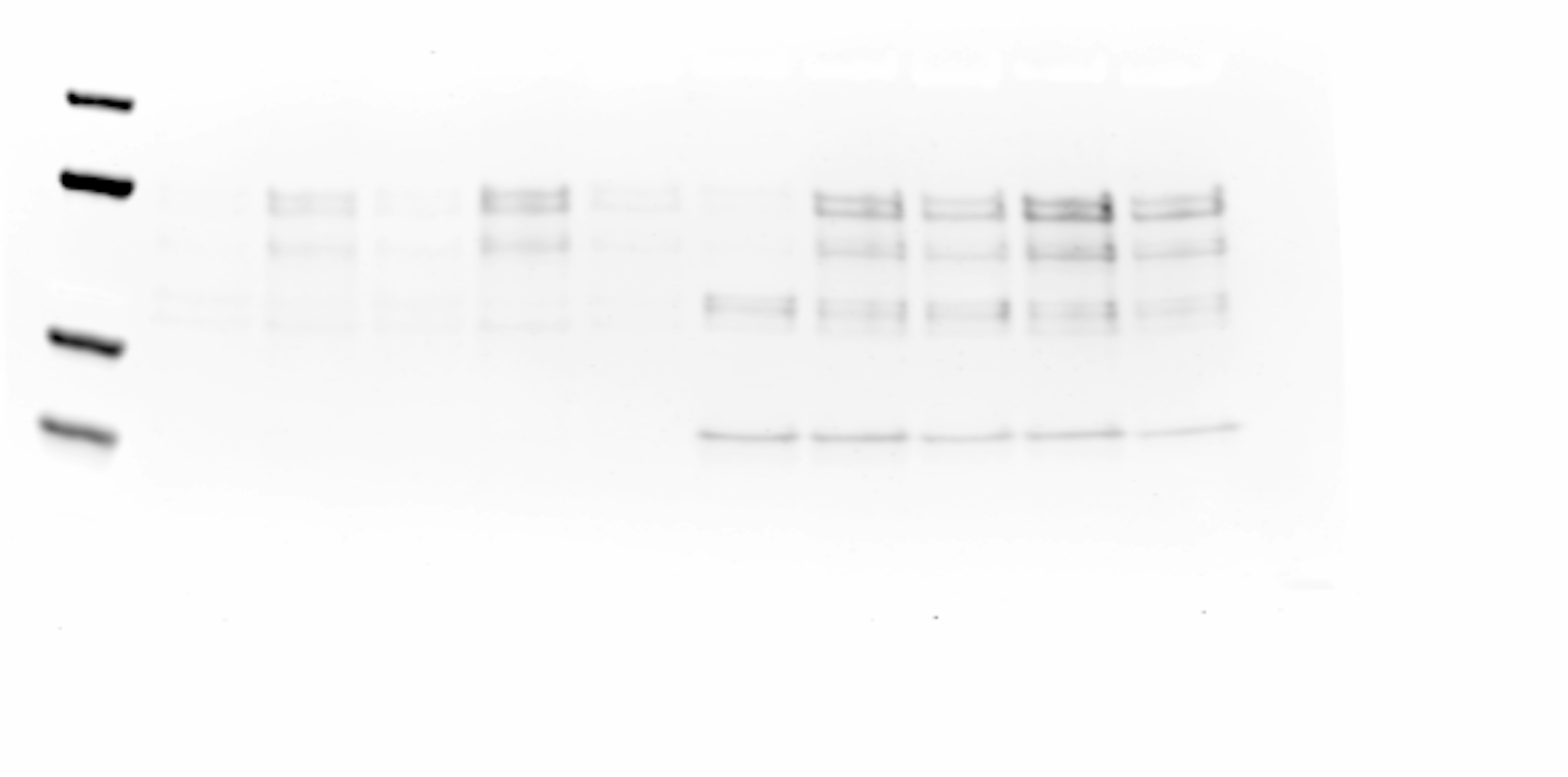

Supplement: Figure 9—source data 1. [file elife-79771-fig9-data1.zip › Figure 9B/rbxpRab10.tif]

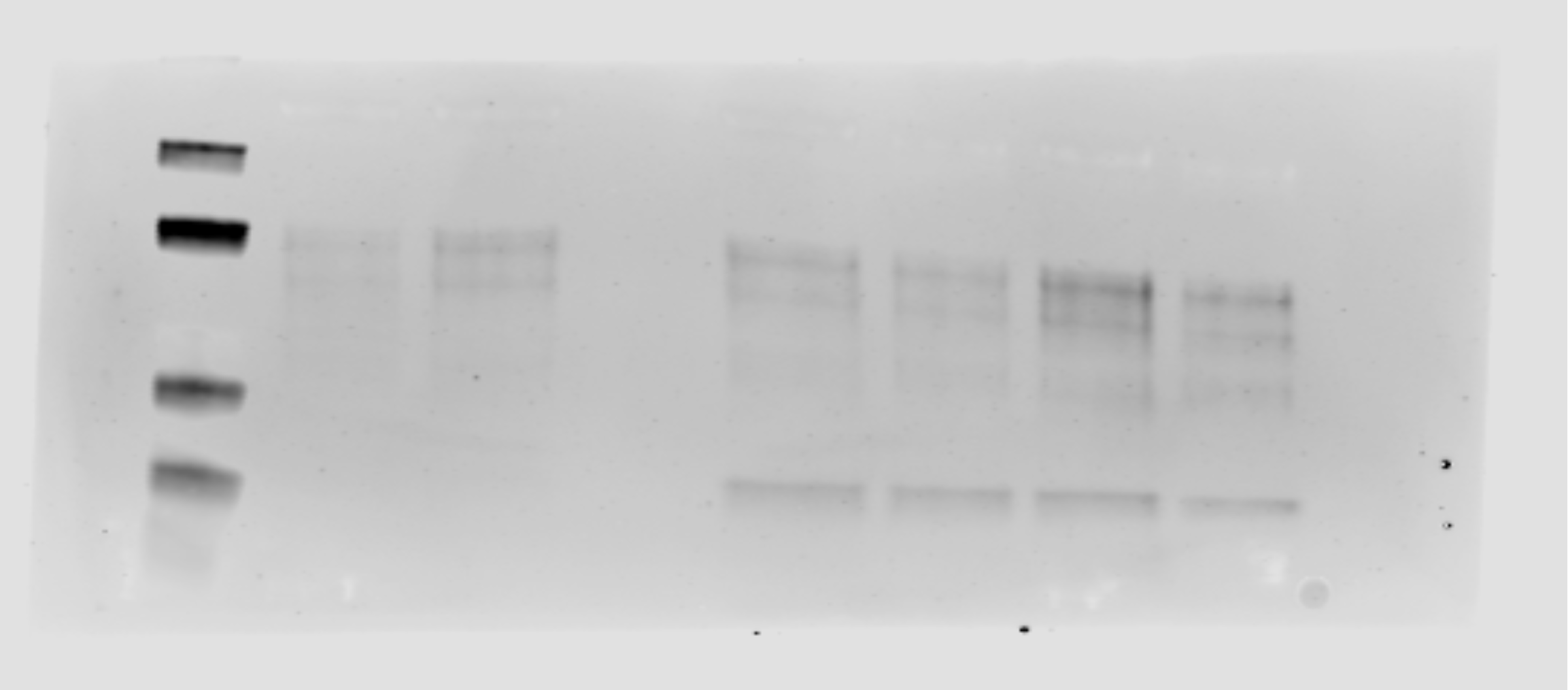

Supplement: Figure 9—source data 1. [file elife-79771-fig9-data1.zip › Figure 9B/rbxpRab10 2.tif]

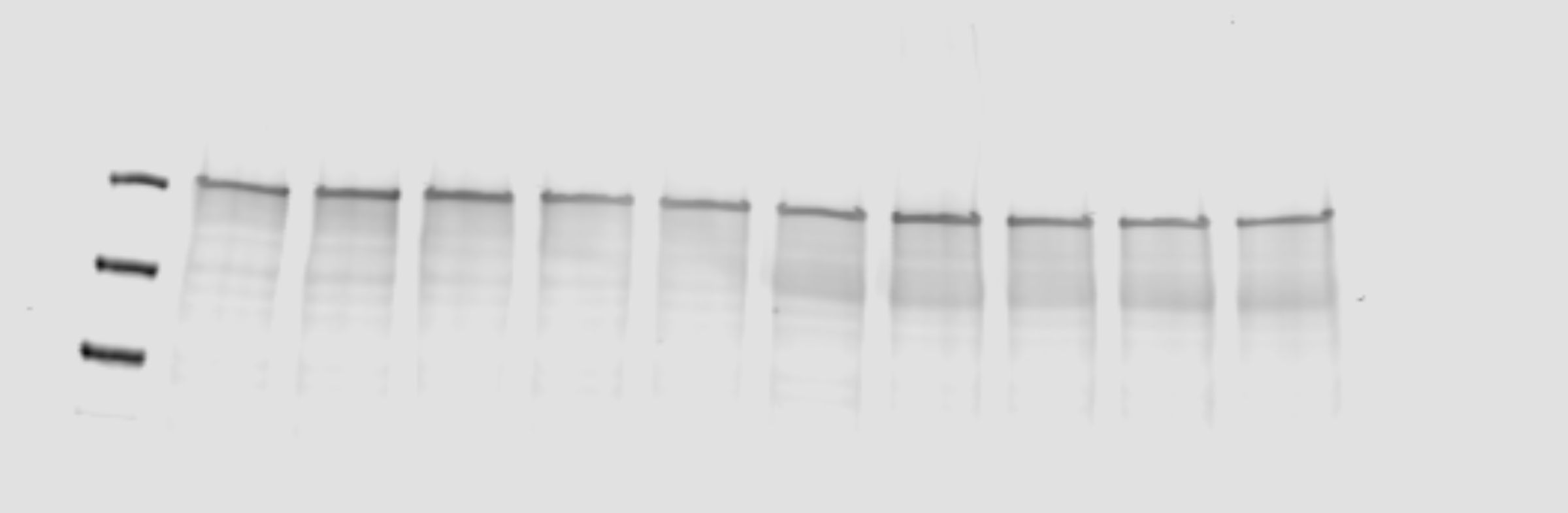

Supplement: Figure 9—source data 1. [file elife-79771-fig9-data1.zip › Figure 9B/msxLRRK2.tif]
