# Supplementary material for: A feed-forward pathway drives LRRK2 kinase membrane recruitment and activation
Source: eLife. 2022 Sep 23;11:e79771. doi: 10.7554/eLife.79771 (PMC9576273; doi:10.7554/eLife.79771)

Fig.3.S4B_HA

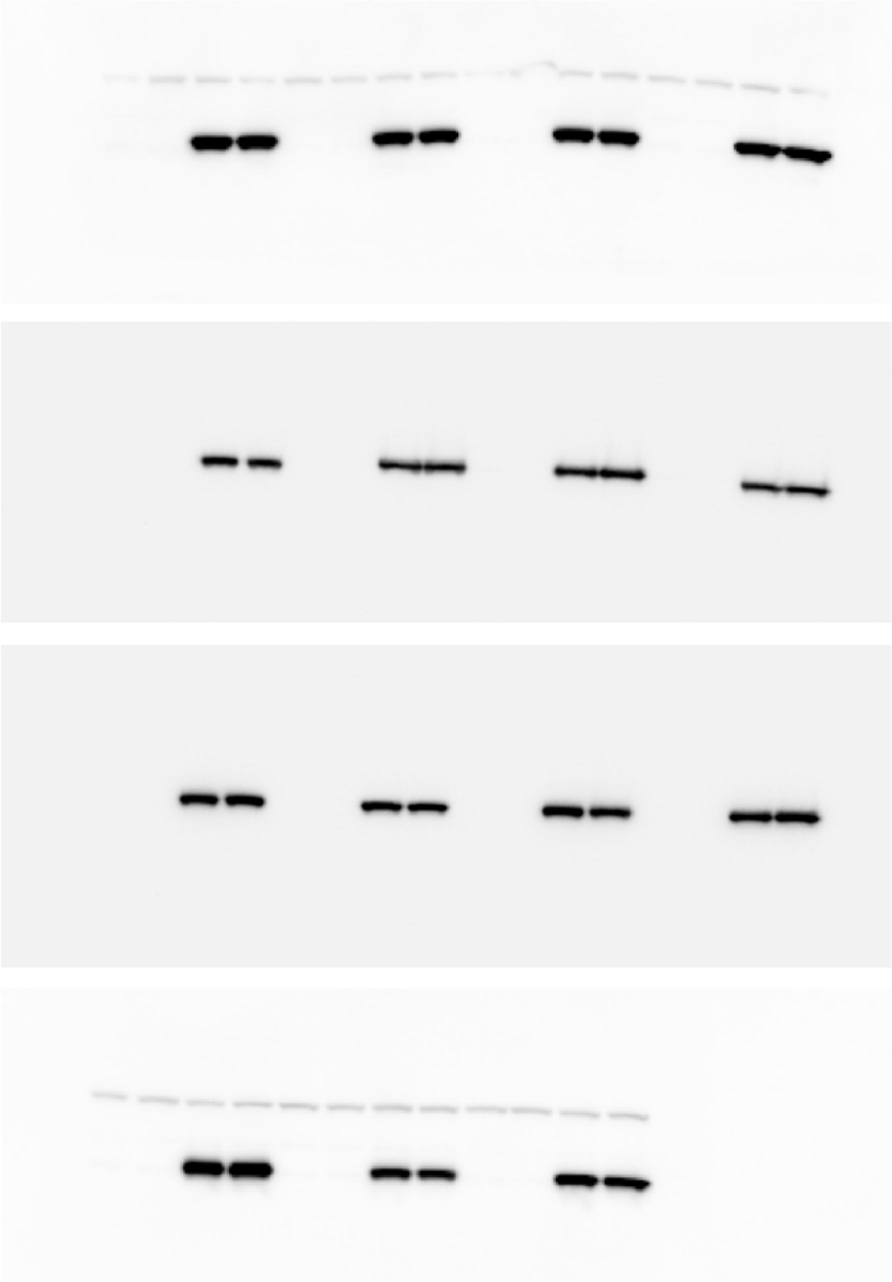

Fig.3.S4B_800(pRab10;pLRRK2)_Low

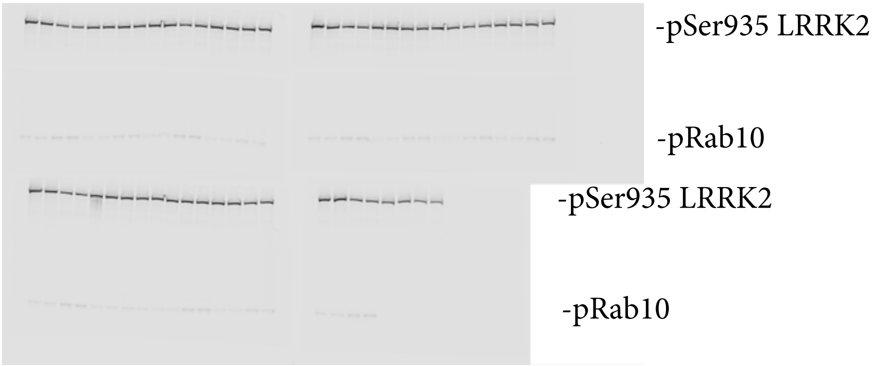

Fig.3.S4B_800(pRab10;pLRRK2)_High

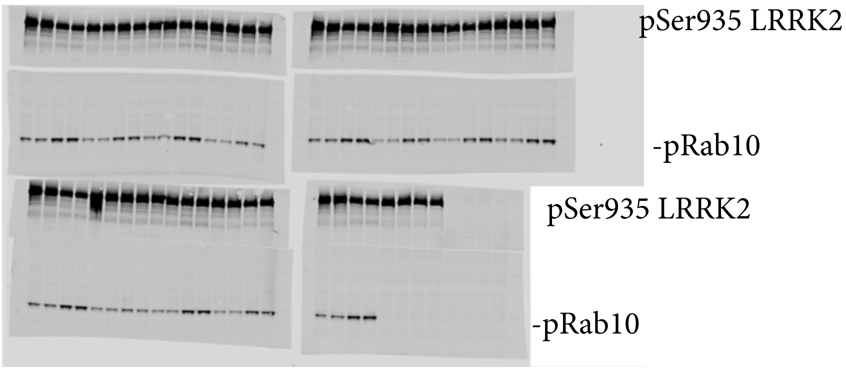

Fig.3.S4B_800(pRab10;pLRRK2)_High

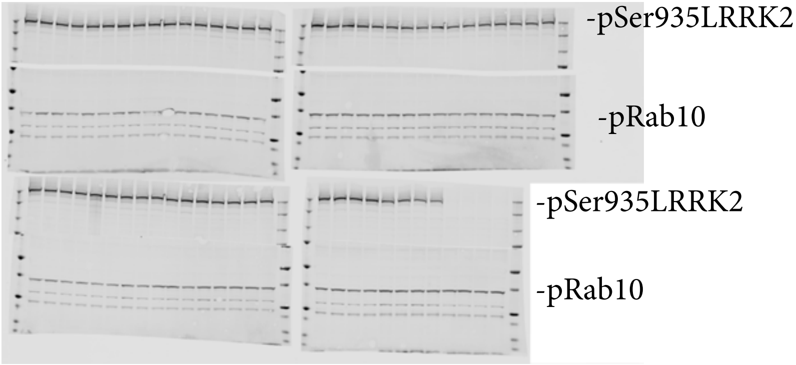

Fig.3.S4B_800(pRab10;pLRRK2)_High

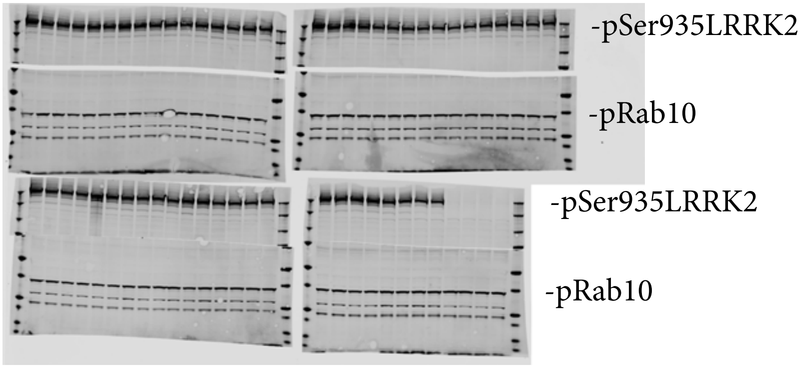

Fig.3.S4A_HA

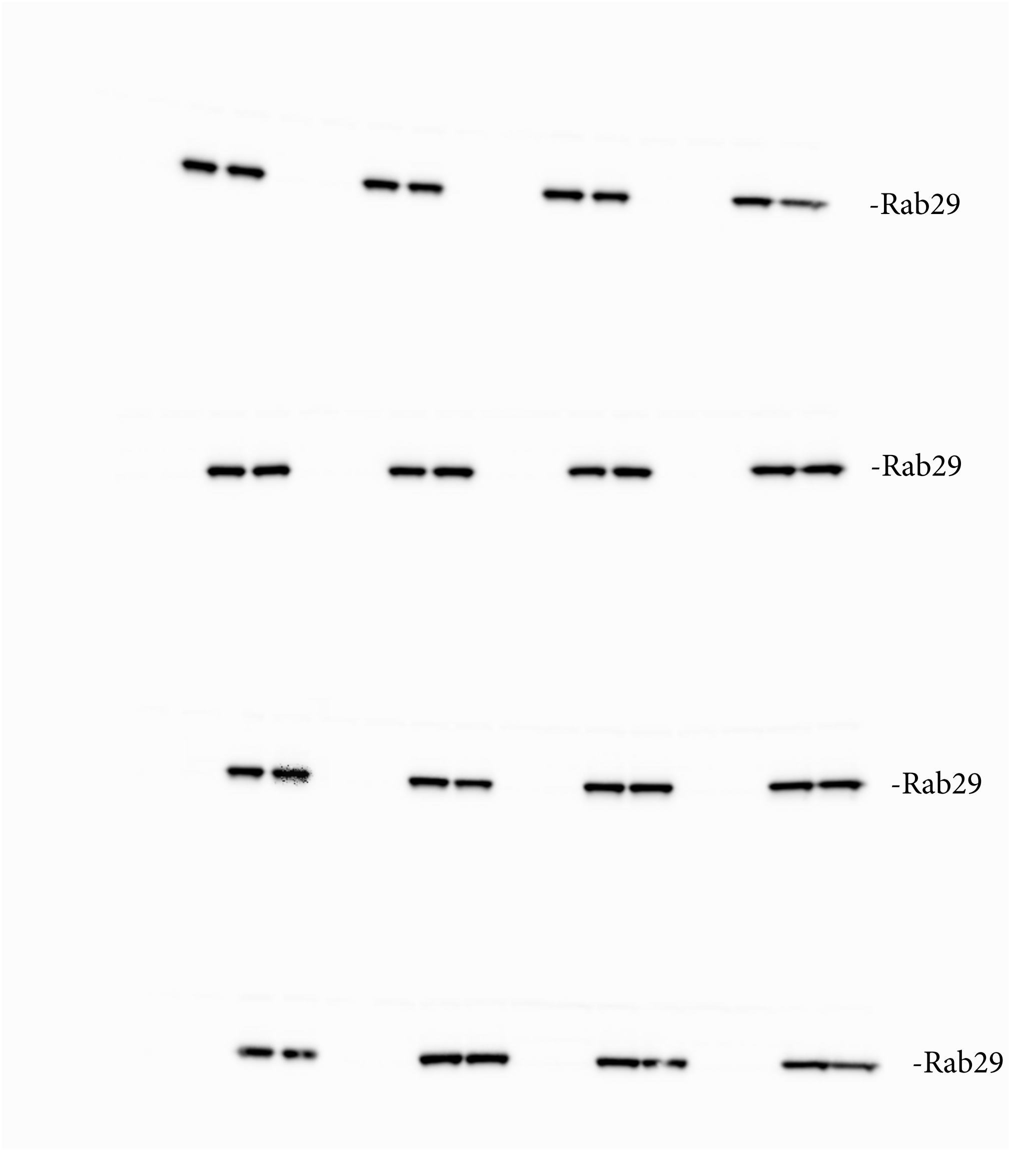

Fig.3.S4A_800(pRab10;pLRRK2)_Low

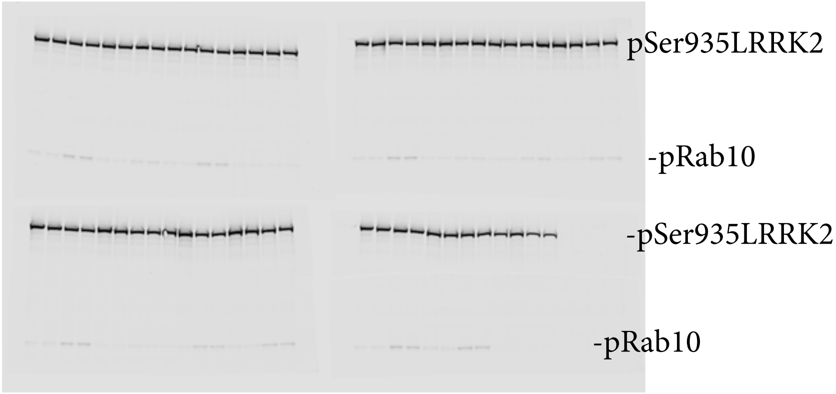

Fig.3.S4A_800(pRab10;pLRRK2)_Low

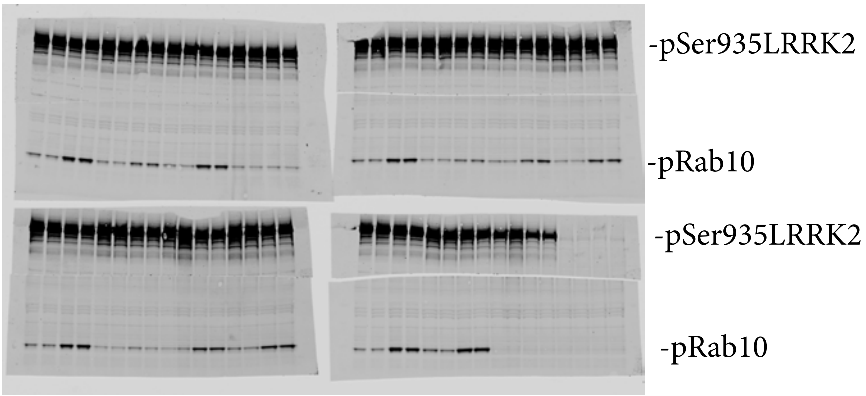

Fig.3.S4A_800(pRab10;pLRRK2)_High

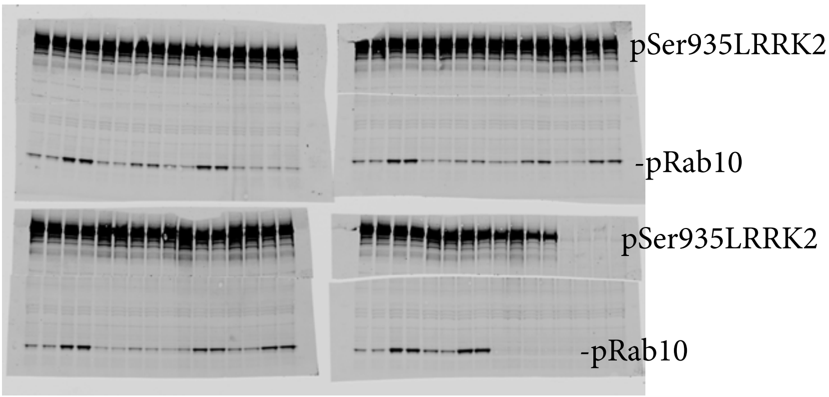

Fig.3.S4A_700(tRab10;tLRRK2)_Low

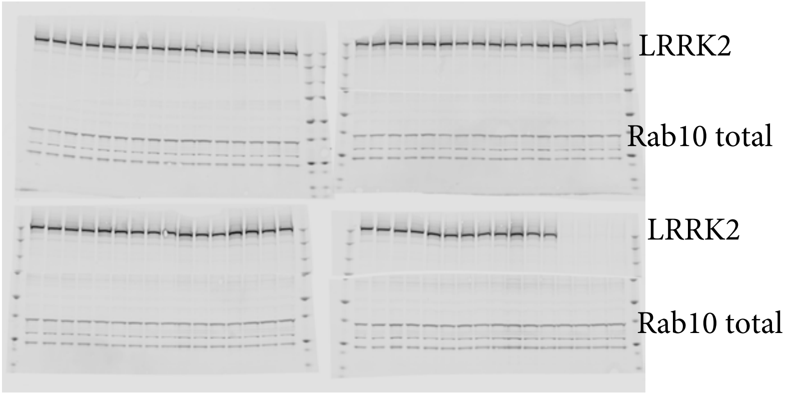

Fig.3.S4A_700(tRab10;tLRRK2)_High

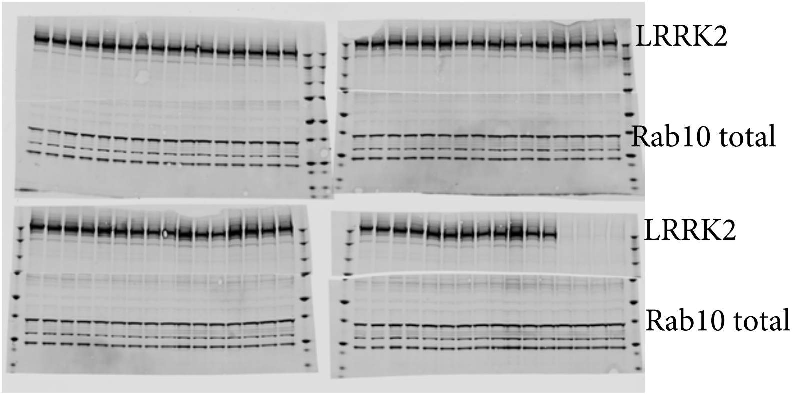

Supplement: Figure 3—figure supplement 4—source data 2. [file elife-79771-fig3-figsupp4-data2.pdf]
