# Supplementary material for: A feed-forward pathway drives LRRK2 kinase membrane recruitment and activation
Source: eLife. 2022 Sep 23;11:e79771. doi: 10.7554/eLife.79771 (PMC9576273; doi:10.7554/eLife.79771)

Fig. 7A,B Experiment 1

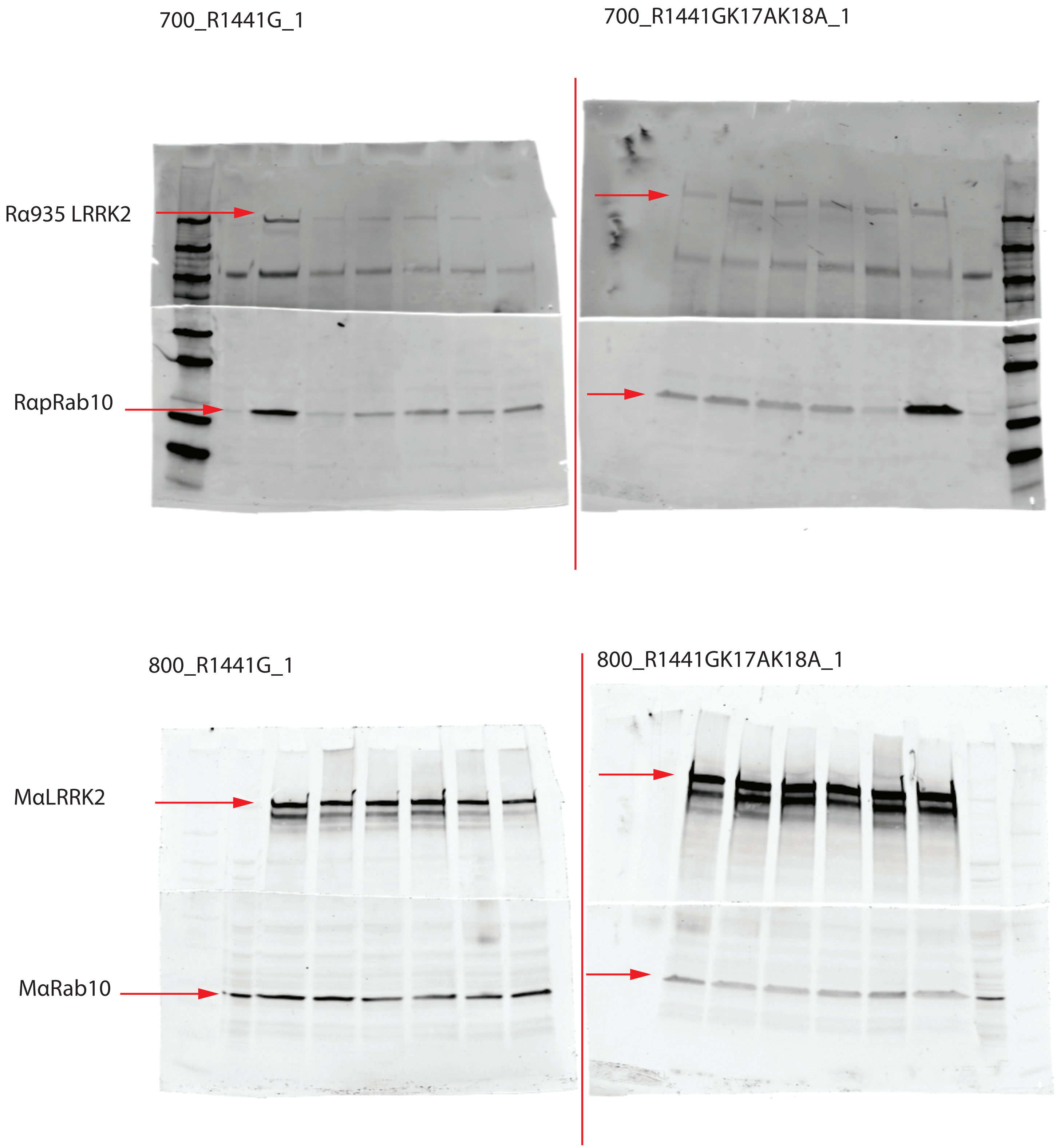

Fig. 7A,B Experiment 2

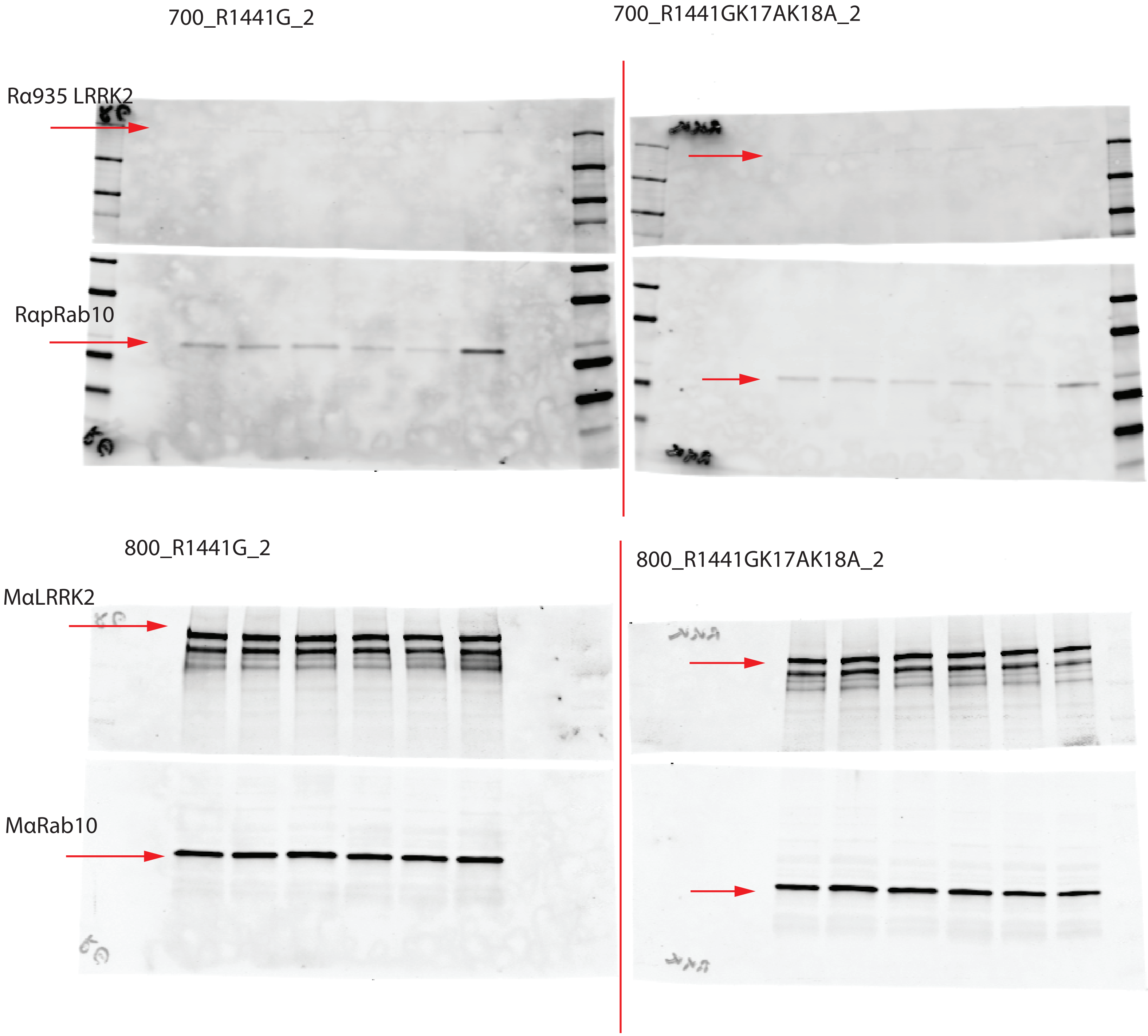

Fig. 7A,B Experiment 3

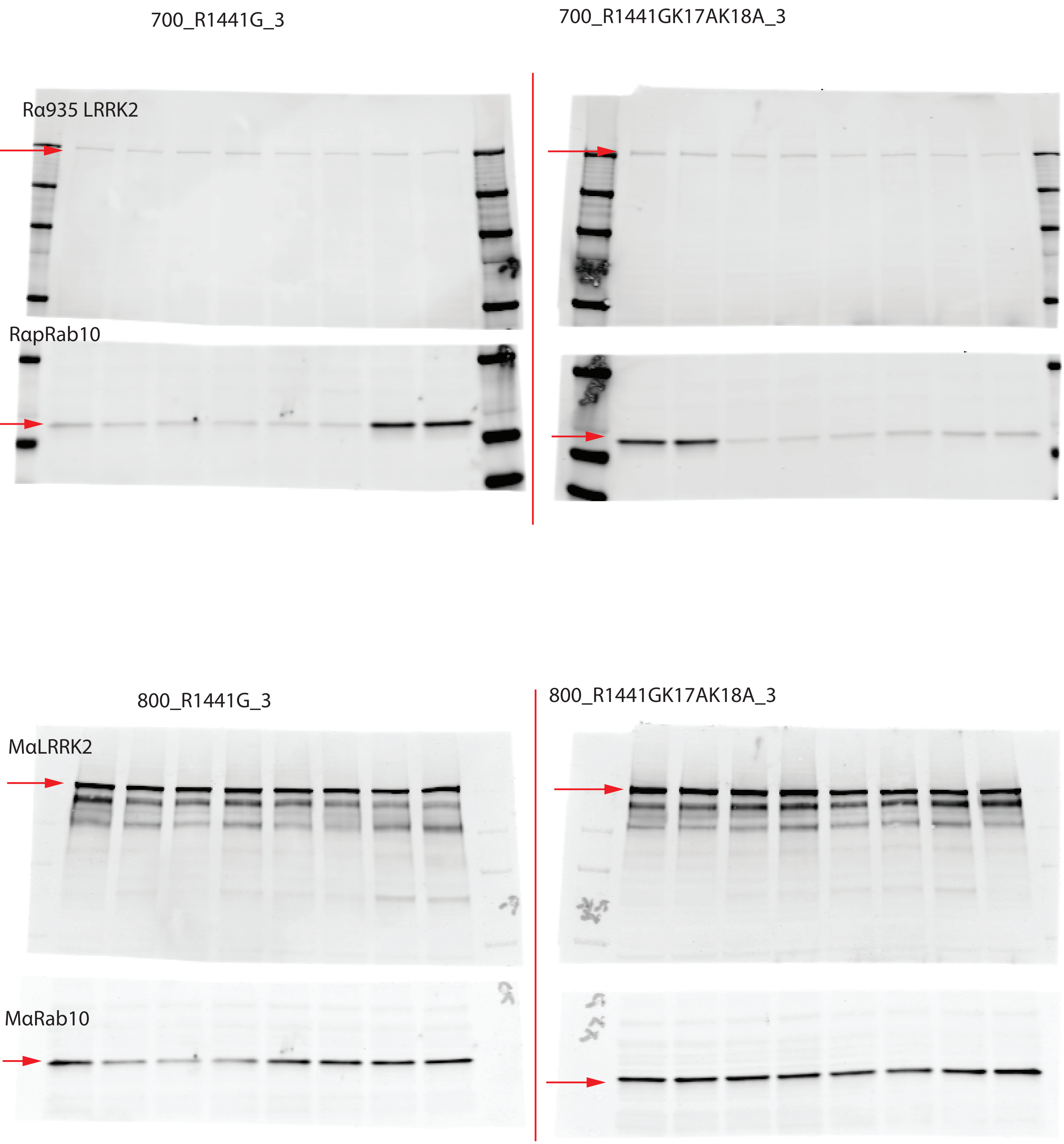

Fig. 7C,D Experiment 1

700_LRRK2_1

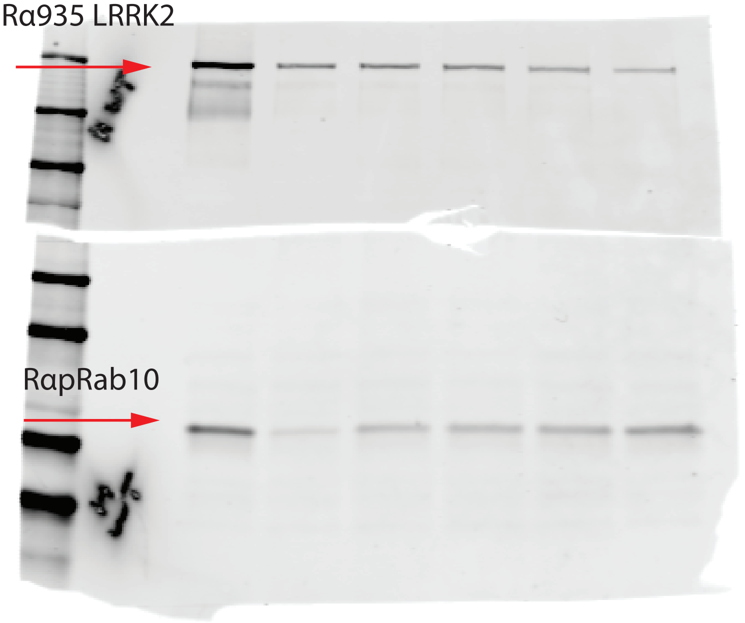

700_K17AK18A_1

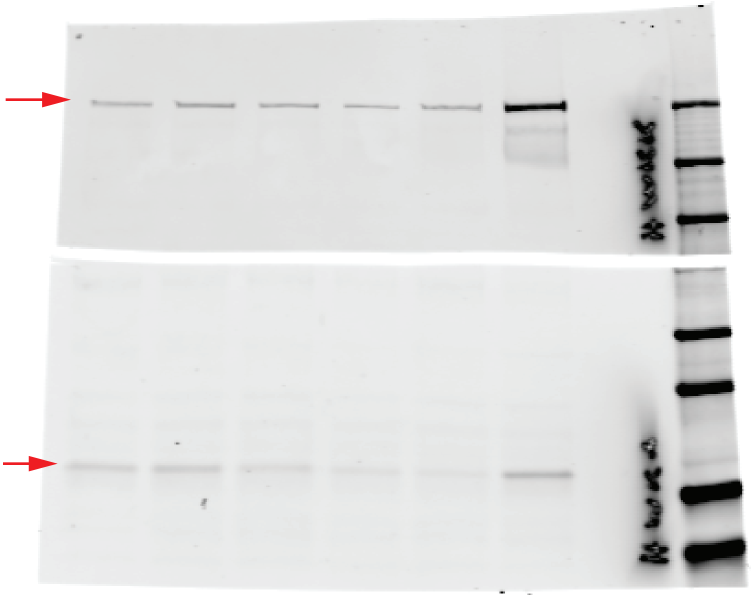

800_LRRK2_1

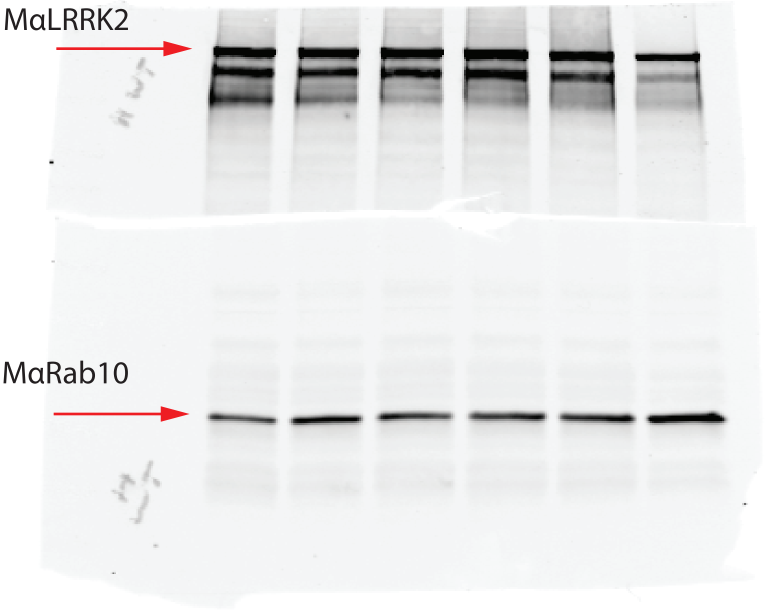

800_K17AK18A_1

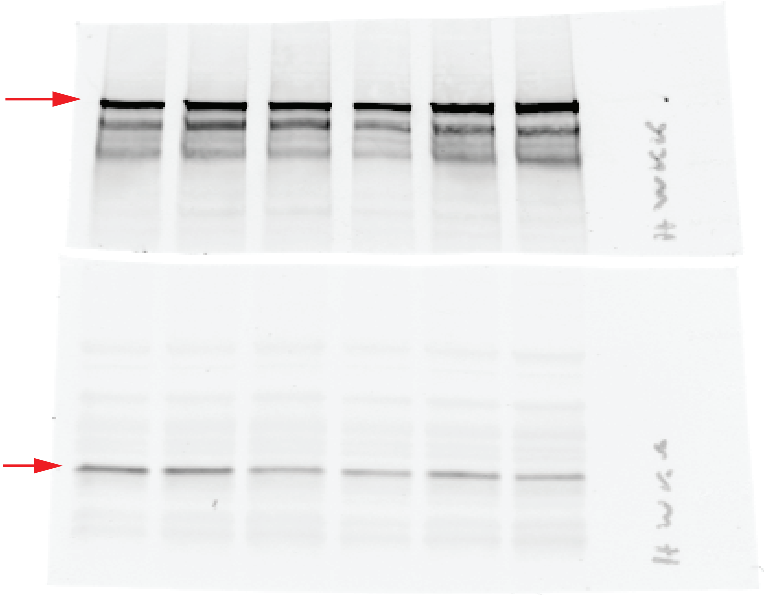

Fig. 7C,D Experiment 2

700_LRRK2_2

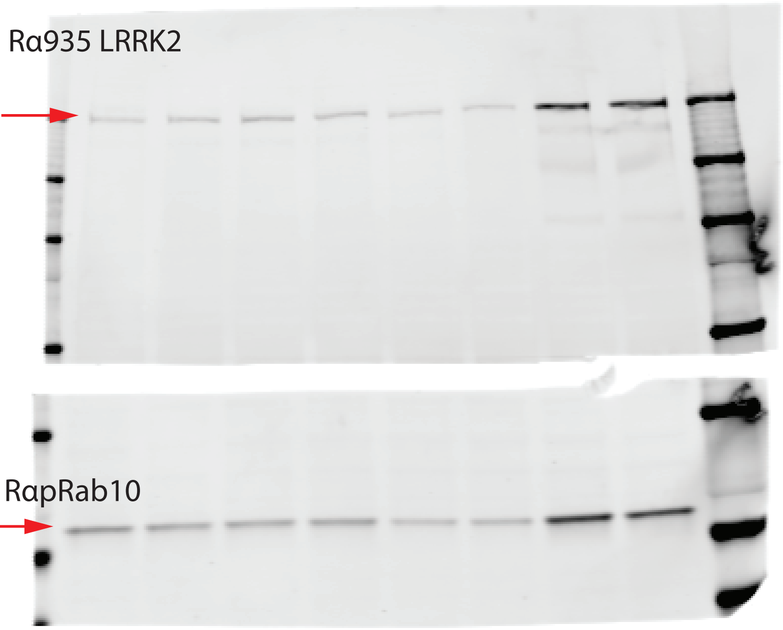

700_K17AK18A_2

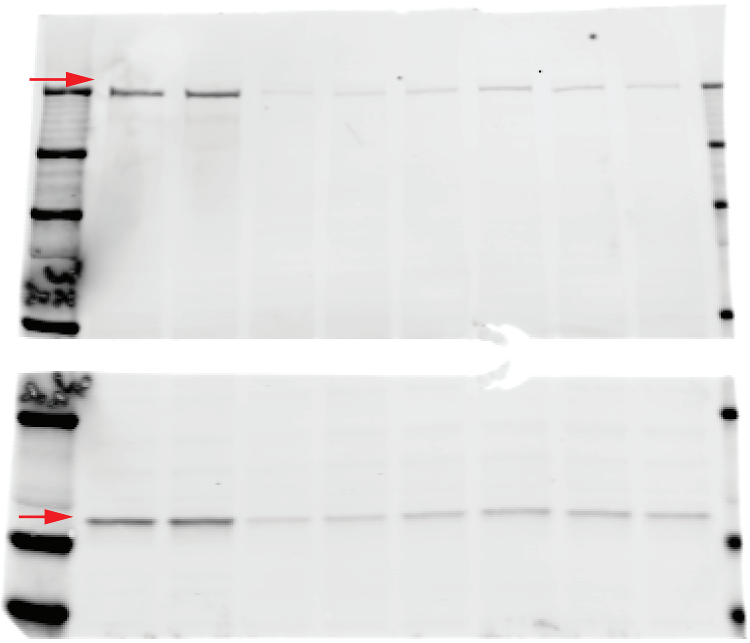

800_LRRK2_2

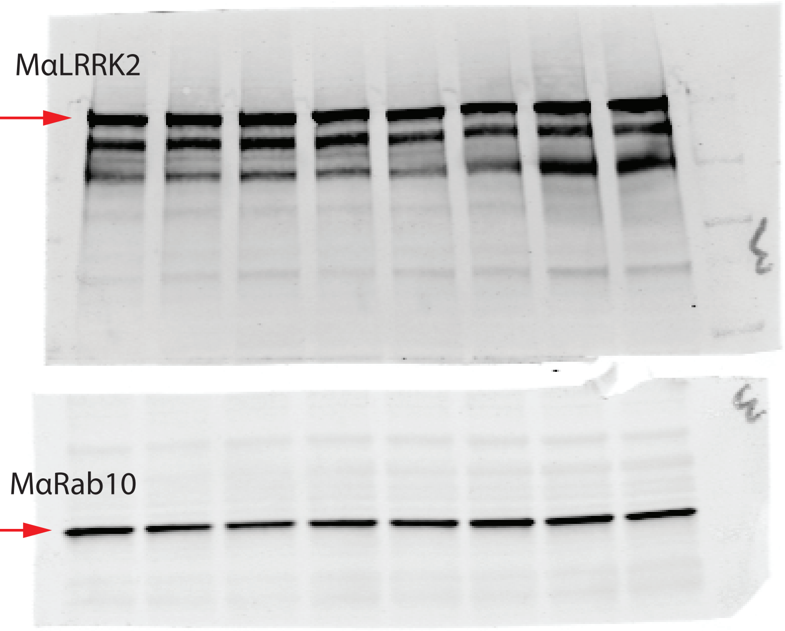

800_K17AK18A_2

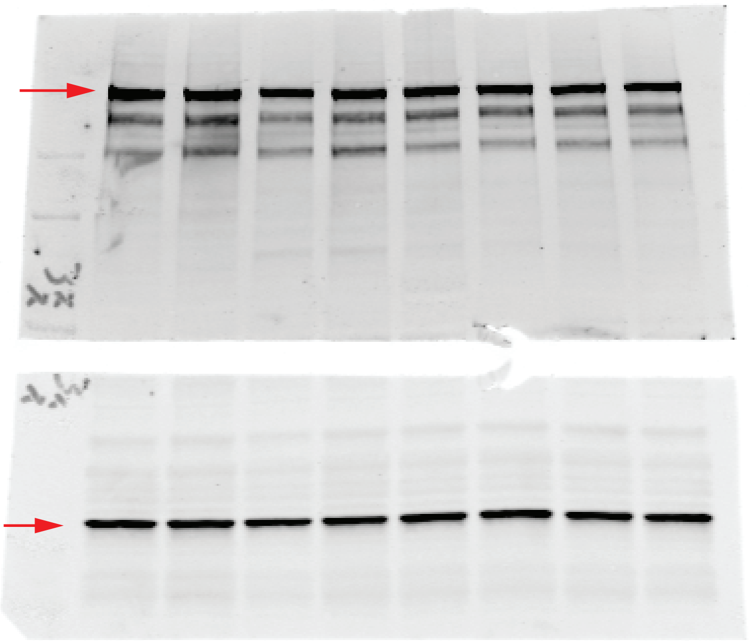

Fig. 7C,D Experiment 3

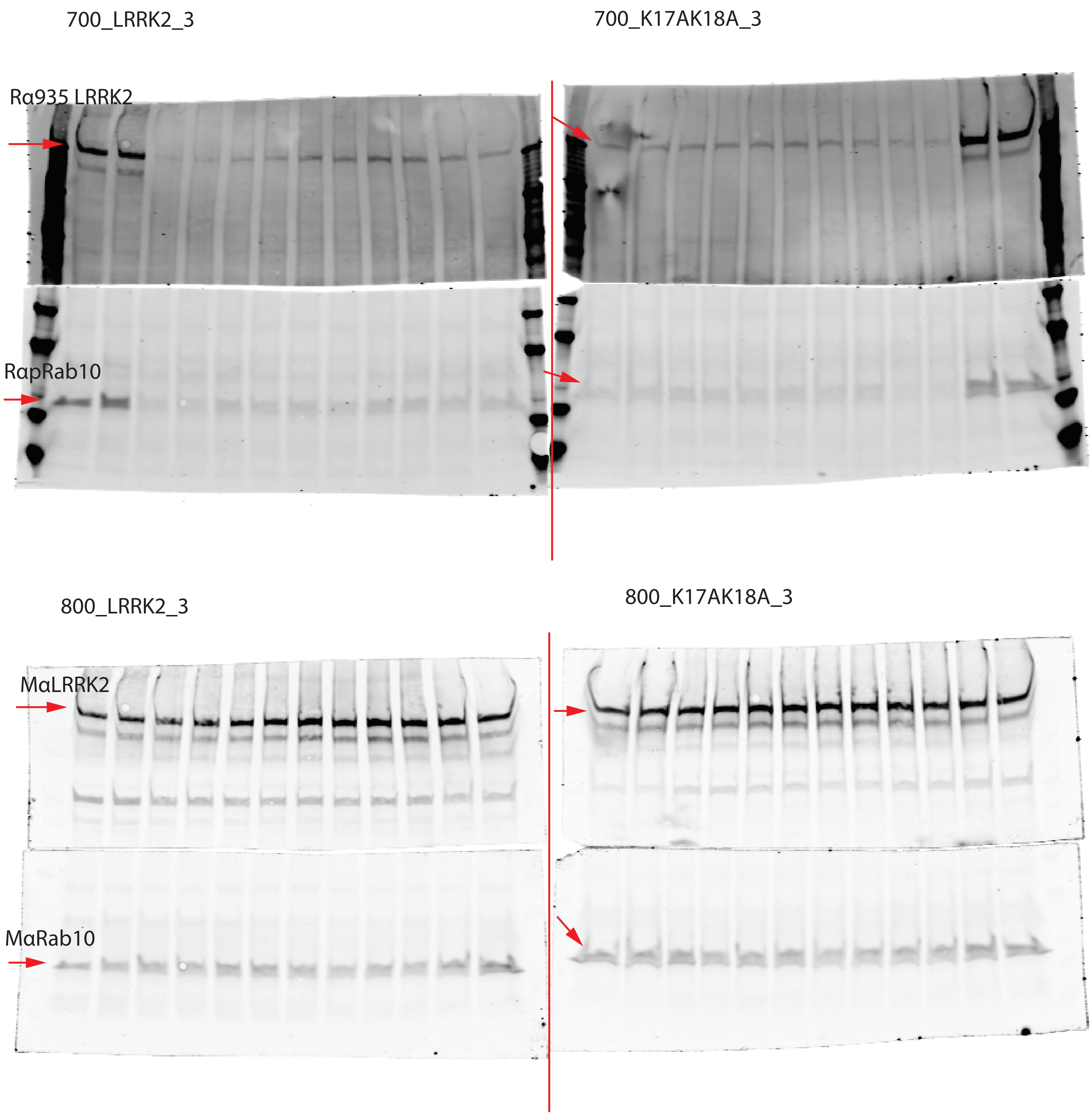

Supplement: Figure 7—source data 2. [file elife-79771-fig7-data2.pdf]
