# Supplementary material for: A feed-forward pathway drives LRRK2 kinase membrane recruitment and activation
Source: eLife. 2022 Sep 23;11:e79771. doi: 10.7554/eLife.79771 (PMC9576273; doi:10.7554/eLife.79771)

Figure 9A

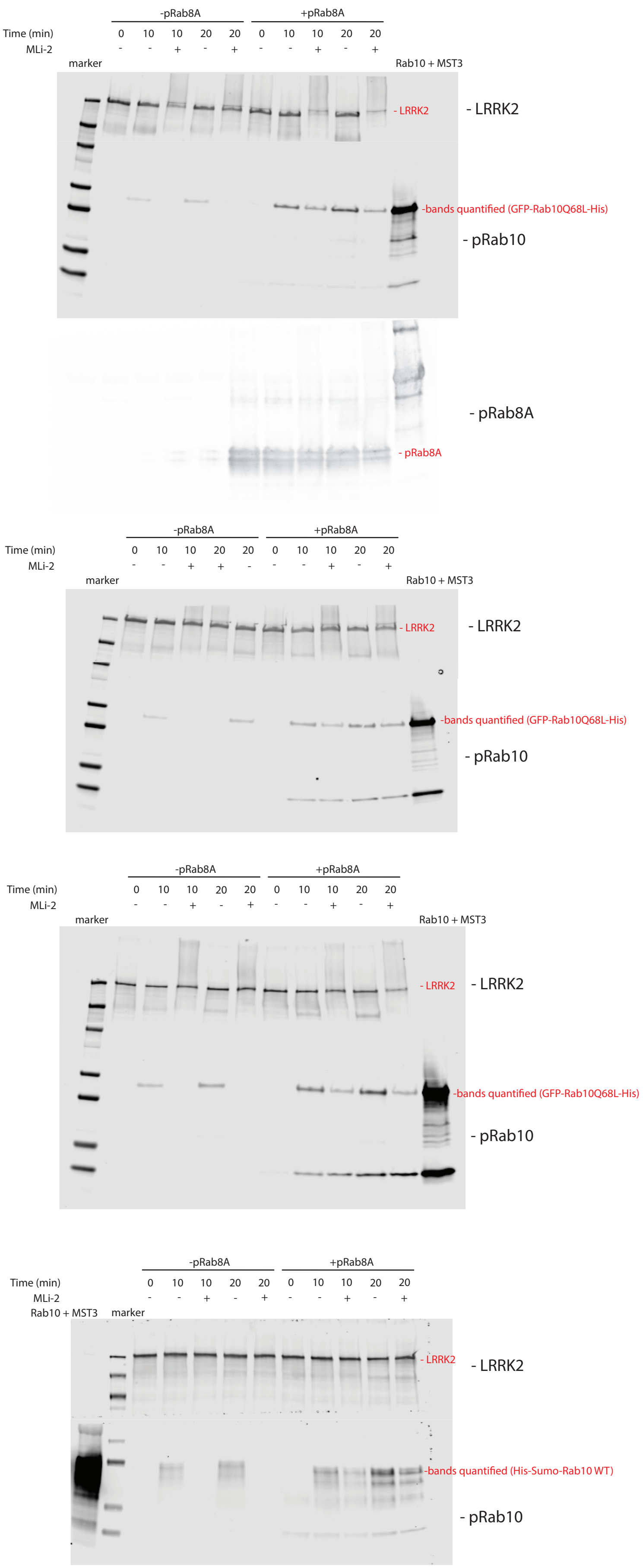

Figure 9B

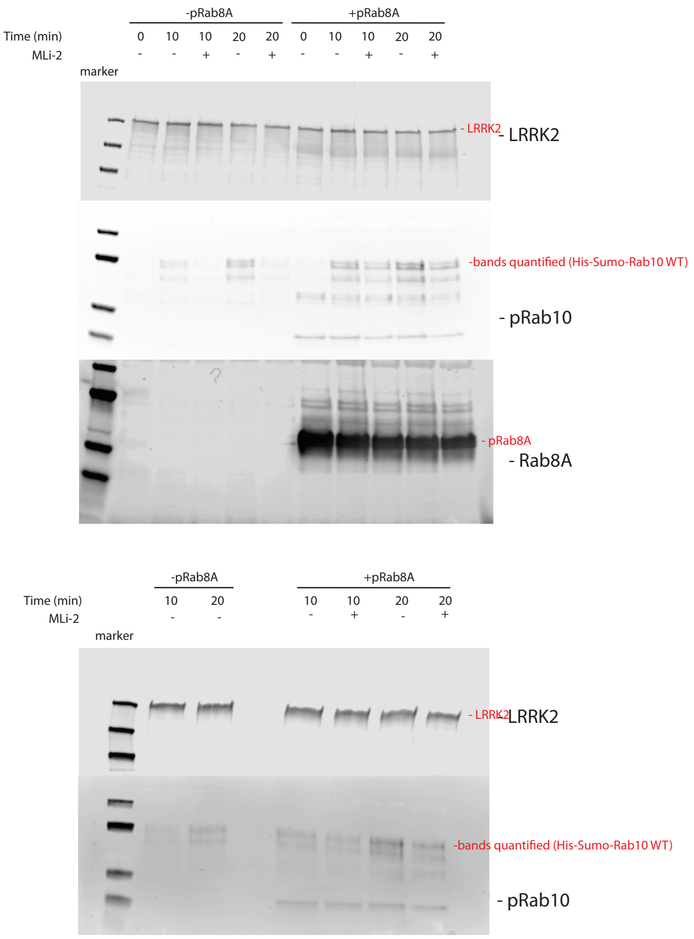

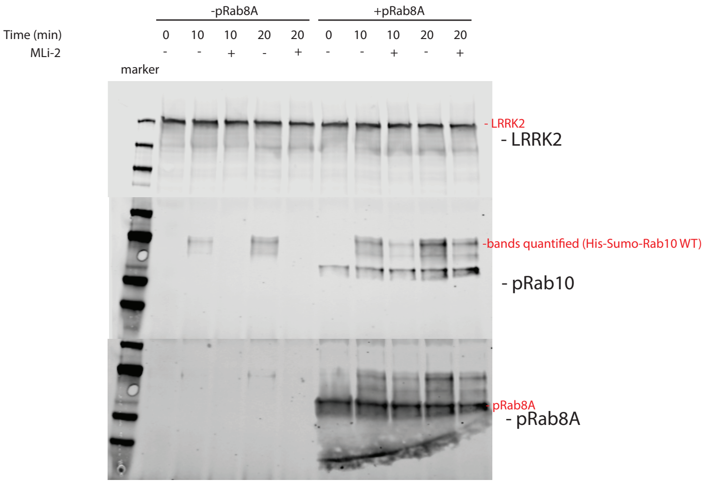

Supplement: Figure 9—source data 2. [file elife-79771-fig9-data2.pdf]
